# Immersion Bioprinting of Tumor Organoids in Multi-Well Plates for Increasing Chemotherapy Screening Throughput

**DOI:** 10.3390/mi11020208

**Published:** 2020-02-18

**Authors:** Erin Maloney, Casey Clark, Hemamylammal Sivakumar, KyungMin Yoo, Julio Aleman, Shiny A. P. Rajan, Steven Forsythe, Andrea Mazzocchi, Adrian W. Laxton, Stephen B. Tatter, Roy E. Strowd, Konstantinos I. Votanopoulos, Aleksander Skardal

**Affiliations:** 1 Department of Biomedical Engineering, Cornell University, Ithaca, NY 14853, USA; eem95@cornell.edu; 2 Wake Forest Institute for Regenerative Medicine, Wake Forest School of Medicine, Winston Salem, NC 27101, USA; cuvan94@vt.edu (C.C.); kmyoo@wakehealth.edu (K.Y.); scrudle93@gmail.com (J.A.); shinyrajan88@gmail.com (S.A.P.R.); sforsyth@wakehealth.edu (S.F.); amazzocc@wakehealth.edu (A.M.); 3 Virginia Tech-Wake Forest School of Biomedical Engineering and Sciences, Winston-Salem, NC 27101, USA; 4 Department of Biomedical Engineering, The Ohio State University, Columbus, OH 43210, USA; sugavanamsivakumar.1@osu.edu; 5 The Ohio State University Comprehensive Cancer Center, Ohio State University Wexner Medical Center, Columbus, OH 43420, USA; 6 Department of Cancer Biology, Wake Forest School of Medicine, Winston Salem, NC 27101, USA; 7 Comprehensive Cancer Center at Wake Forest Baptist Medical Center, Winston-Salem, NC 27157, USA; alaxton@wakehealth.edu (A.W.L.); statter@wakehealth.edu (S.B.T.); rstrowd@wakehealth.edu (R.E.S.); kvotanop@wakehealth.edu (K.I.V.); 8 Department of Neurosurgery, Wake Forest Baptist Medical Center, Winston-Salem, NC 27157, USA; 9 Department of Neuroscience, Wake Forest Baptist Medical Center, Winston-Salem, NC 27157, USA; 10 Department of Surgery–Oncology, Wake Forest Baptist Medical Center, Winston-Salem, NC 27157, USA

**Keywords:** bioprinting, bioink, extracellular matrix, cancer, organoid, drug screening, personalized medicine

## Abstract

The current drug development pipeline takes approximately fifteen years and $2.6 billion to get a new drug to market. Typically, drugs are tested on two-dimensional (2D) cell cultures and animal models to estimate their efficacy before reaching human trials. However, these models are often not representative of the human body. The 2D culture changes the morphology and physiology of cells, and animal models often have a vastly different anatomy and physiology than humans. The use of bioengineered human cell-based organoids may increase the probability of success during human trials by providing human-specific preclinical data. They could also be deployed for personalized medicine diagnostics to optimize therapies in diseases such as cancer. However, one limitation in employing organoids in drug screening has been the difficulty in creating large numbers of homogeneous organoids in form factors compatible with high-throughput screening (e.g., 96- and 384-well plates). Bioprinting can be used to scale up deposition of such organoids and tissue constructs. Unfortunately, it has been challenging to 3D print hydrogel bioinks into small-sized wells due to well–bioink interactions that can result in bioinks spreading out and wetting the well surface instead of maintaining a spherical form. Here, we demonstrate an immersion printing technique to bioprint tissue organoids in 96-well plates to increase the throughput of 3D drug screening. A hydrogel bioink comprised of hyaluronic acid and collagen is bioprinted into a viscous gelatin bath, which blocks the bioink from interacting with the well walls and provides support to maintain a spherical form. This method was validated using several cancerous cell lines, and then applied to patient-derived glioblastoma (GBM) and sarcoma biospecimens for drug screening.

## 1. Introduction

Many drugs fail to make the transition from preclinical to clinical studies and do not prove to be effective in phase 2 studies despite promising reports in preclinical models. Likewise, many drugs are discarded based on lack of activity in preclinical models but may have activity in vivo. This contributes to the inefficiency of drug development and requires better models that more accurately recapitulate the host disease state including models that reflect drug resistance or particular mutations as seen in patients [1,2]. Failure to respond to treatment as well as drug resistance to anti-cancer drugs are critical challenges in clinical oncology that can be ascribed to factors like genetic mutation [3,4,5], oncogenic amplification [6,7], and other changes in the tumor cell machinery that result in changes in the uptake capability, metabolism of drugs, and removal of drugs and drug metabolites from the cells. Because of such dynamic evolving factors in cancer cells, resistance to drugs can arise quickly and has been documented for most commonly prescribed anti-cancer treatments, often with strikingly high rates, but varying widely between patients. Such variable response to therapy is currently addressed through precision medicine (PM) by relating genetic mutations and expression profiles to therapeutic options. However, in practice, even after the identification of probable targets, modification of a predetermined traditional treatment strategy is still rare given the lack of empirical data demonstrating the efficacy of a targeted therapy. Moreover, despite its potential, the clinical efficacy of PM-driven treatment is typically poor [8]. Although the statistics vary with disease, in general, only 11% of patients entered into PM programs experience a change to an alternative therapeutic; of those patients who do receive an altered treatment, only 3% experience what could be considered a significant improvement in outcome compared to traditional therapy [9]. This means that current PM tools used to predict and drive treatment decisions are not much better, if any, than the standard approach and thus needs to be improved.

Two dimensional (2D) cell culture techniques have provided biological models that have resulted in numerous scientific advances and discoveries. However, these culture techniques fail to accurately represent the three dimensional (3D) biology of the in vivo tumor microenvironment [10]. Cells placed into 2D environments experience completely foreign topographies, substrate mechanical properties, cell–cell and cell–matrix interactions, and diffusion kinetics of nutrients and oxygen in comparison to bioengineered 3D models. Instead, 2D cell culture conditions, such as plastic surfaces, can induce significant changes in cells at the genetic and phenotypic level, resulting in experimental results that may not be truly representative of in vivo biology [11,12]. In earlier studies, members of our team demonstrated that when metastatic colorectal cancer cells were maintained in 2D culture, they took on a clear epithelial phenotype, but when incorporated a 3D organoid environment they quickly adopted a phenotype that appeared both mesenchymal and metastatic, which was accurately reflective of their in vivo tumor of origin [13,14]. Numerous cell line-based tumor organoids and tumor-on-a-chip models have been created by our group and others that show the importance of the 3D microenvironment.

This discovery has led to the creation of 3D organoid models created from patient tumors, filling a critical scientific gap, to facilitate drug screening studies that can provide patient-specific empirical data to better predict a patient’s drug response. Typically, we combine organ micro-engineering [15,16], often with microfluidics [13,14,17,18,19], with tissue/tumor-inspired extracellular matrix (ECM) biomaterials, to produce more biologically relevant organoid models. This 3D organoid biofabrication approach combines multiple facets that support improved recapitulation of certain in vivo conditions, including architectures in 3D rather than 2D, cell–cell and cell–ECM interactions, circulatory systems if relevant, and even integration of a subset of immune cells should the application demand it. Our lab has succeeded in creating such patient-derived tumor organoids (PTOs) and tumor-on-a-chip models from lung, mesothelioma, melanoma, colorectal, appendiceal, glioblastoma, sarcoma, and several rare tumors, and have deployed these models in chemotherapy and immunotherapy drug screens [20,21,22,23]. While certainly useful for disease modeling, mechanistic study, and drug development, we have also used such models in a diagnostic sense to influence therapy, which is perhaps the ultimate goal.

There has been a challenge in deploying PTOs in high-throughput formats and maintaining consistency. Most current PTO systems are inconsistent in terms of size, geometry, and cell density; have poor take rates, fail to form spheroids in hanging drop scenarios, are not amenable to high-throughput formats, and rely on Matrigel-induced self-organization/differentiation. ECM bioinks and bioprinting offer a potential solution.

Bioprinting is a versatile technology with potential for a wide variety of applications in regenerative medicine and tissue engineering. Bioprinting can be described as computer-controlled additive biofabrication with the potential to build or pattern viable organ-like or tissue structures in 3 dimensions (3D) using cells and biomaterials. To date, complete human-sized organs have not been printed, but this remains the ultimate goal. Currently, bioprinted constructs have been implanted in animals [24], and small-scale bioprinted tissue constructs, or “organoids”, are being implemented in a number of applications. These include disease modeling, drug and toxicology screening, and, recently, personalized medicine in cancer [17,25,26,27,28,29,30,31]. One of the major problems that the field of bioprinting, is that few advances have occurred in regard to approaches to the printing process itself, or generation of novel, more user-friendly bioinks. Unfortunately, many bioprinting studies are somewhat repetitive—falling back on traditional biomaterials and their crosslinking approaches, which were never developed to be bioprinted or to accurately represent the complexities of the native ECM. Our laboratory has over a decade of experience in developing ECM-derived bioinks with inherent characteristics to improve printability [29,30,31]. These have ranged from multi-step crosslinking reaction bioinks to, recently, thixotropic bioinks that allow for the simple introduction of cells within the bioink precursors and maintenance of the mechanical properties necessary to ensure extrusion, while still supporting deposition of free-standing 3D structures [17,27,28,32]. We have recently been working to create modular, defined bioinks with key ECM components that boost cell viability, phenotype, and function of healthy primary cells and patient-derived tumor cells [25,32,33,34]. Additionally, in recent years we have seen various laboratories pushing the bioprinting field by developing novel methodologies of bioprinting that are not simply inkjet and extrusion bioprinting. This includes laser-induced forward transfer bioprinting [35] and freeform reversible embedding of suspended hydrogels (FRESH) bioprinting [36,37] (from which we certainly have taken inspiration), among others. Advances in methodologies such as these, paired with improved bioinks developed specifically for bioprinting, are opening up new opportunities for bioprinting-based applications.

Here, we describe a 3D bioprinting approach—“immersion bioprinting”—which aims to mitigate the limitations that have plagued tumor organoid systems as we described above. Realization of this technology that can fabricate PTOs in a consistent and high-throughput fashion will provide a valuable ex vivo/in vitro tool that can be deployed for many subsequent studies, including target discovery, mechanistic investigation of tumor biology, drug development, and personalized drug screens to aid in treatment selection in the clinic.

## 2. Materials and Methods

### 2.1. Hydrogel Bioink Formulations and Preparation

The collagen–hyaluronic acid (HA) bioink was made using methacrylated collagen and thiolated HA and was prepared using the following steps. Methacrylated collagen type I (Coll-MA, Advanced BioMatrix, Carlsbad, CA, USA) was reconstituted with 20 mM acetic acid according to the manufacturer’s protocol to produce a concentration of 6 mg/mL. Immediately prior to use, 1 mL Coll-MA was neutralized with 85 µL of neutralization buffer (Advanced BioMatrix) and mixed with thiolated and heparinized HA (Heprasil^®^, ESI-BIO, Alameda, CA, USA). The HA component was first dissolved in sterile water containing 0.05% w/v of the photoinitiator 2-Hydroxy-4′-(2-hydroxyethoxy)-2-methylpropiophenone (Sigma, St. Louis, MO, USA) to make a solution 1% w/v HA. The final hydrogel was comprised of Coll-MA and HA at a ratio of 1:3, in which the thiol groups of the HA and the methacrylate groups of the Coll-MA were crosslinked. As a bioink, typically this formulation is allowed to partially crosslink through thiol-methacrylate crosslinking, resulting in a soft hydrogel that can be further crosslinked by methacrylate-methacrylate photopolymerization at a later point after printing.

As a comparison, the HyStem–HP hydrogel product was employed and prepared as previously described [38]. Briefly, a thiolated HA component (Heprasil^®^), a thiolated gelatin component Gelin-S^®^), and polyethylene glycol diacrylate crosslinker (PEGDA, Extralink^®^) were dissolved separately in sterile water containing 0.1% w/v of the photoinitiator 2-Hydroxy-4′-(2-hydroxyethoxy)-3-methylpropiophenone (Sigma) to make solutions containing 1% w/v. These solutions were then mixed together in a 2:2:1 ratio by volume, respectively, for immediate testing.

### 2.2. Immersion Bioprinting Evaluation

Two commercially available bioprinters were employed to evaluate the compatibility of the collagen–HA hydrogel and the HyStem–HP hydrogel—the Cellink Incredible bioprinter (Cellink, Boston, MA, USA) and the Allevi Allevi2 bioprinter (Allevi, Philadelphia, PA, USA). First, simple prints were performed to assess the gcode that was written, and the printers’ capability to correctly deposit material in the center of each well of 96-well plates. This was performed simply by using printed water droplets to assess print accuracy. Once gcode–printer compatibility was established, hydrogel-only constructs were bioprinted using custom-written gcode to drive the bioprinters (Supplementary File S1). The 96-well plates were prepared by filling each well with 150 μL of a 10 mg/mL gelatin solution. Each hydrogel was then deposited by the bioprinter in approximately 20 μL volumes in each well of the 96-well plates. Printing procedures were evaluated largely visually and quantitatively, and the total time of printing was recorded.

Additionally, the rheological properties of the collagen–HA hydrogel and the gelatin immersion baths were assessed to determine whether we were employing optimal printing conditions. The bioink as well as 3 gelatin bath concentrations (5, 10, and 20 mg/mL, stored at 4 °C until printing) were analyzed as previously described [32] using a TA instruments DHR-2 rheometer (TA Instruments, New Castle, DE, USA) with a 25 mm plate and 25 mm 2° cone system with 120 grit sandpaper intimately bonded to the surfaces. This addition mimics a roughened geometry for better adhesion to the bioink, preventing slippage during testing. The rheological test was a simple strain sweep from 1% to 1000% shear strain (γ), during which the storage modulus (*G*′) and the loss modulus (*G*″) of each material were recorded.

### 2.3. Cell Line Culture

For the initial analysis of hydrogel biocompatibility, two common cell lines were employed. Human liver cancer cell line (HepG2, HB-8065; American Type Culture Collection (ATCC), Manassas, VA, USA) and human colorectal cancer epithelial cell line (Caco2, HTB-37; ATCC) were cultured in Dulbecco’s Modified Eagle Medium—High Glucose (4.5 g/L) (DMEM-HG; Lonza, Benicia, CA, USA) supplemented with 10% fetal bovine serum (FBS; Hyclone, Logan, UT, USA), 1% L-glutamine (Hyclone) and 1% penicillin/streptomycin (P/S; Hyclone) at 37 °C with 5% CO_2_. Cells were used at 90% confluence.

### 2.4. Cell Line Organoid Immersion Bioprinting

The cells were trypsinized with 0.05% of trypsin (Hyclone) and counted. Then, collagen–HA hydrogel precursors at a concentration of 10 million cells pTissue constructs of each model cell line (HepG2 and Caco2) were created by suspending ter mL, after which the cell-containing suspensions were drawn into printhead syringes. Prior to cell preparation, 96-well plates were prepared by filling each well with 150 μL of the 10 mg/mL gelatin solution. Well plates were placed at 4 °C until needed. 

Hydrogel–cell organoids were bioprinted using custom-written gcode to drive the bioprinters (Supplementary File S1). The printhead needle traveled into the gelatin bath of each well and deposited approximately 20 μL of cell-laden bioink before moving to another well and repeating. A UV lamp (365 nm, 18 W/cm^2^, Dymax BlueWave 75, Dymax Corporation, Torrington, CT, USA) was then employed to irradiate each well for about 1 s each, initiating the crosslinking reaction between remaining thiol groups and methacrylate groups and between methacrylates groups themselves. Following crosslinking, well plates were transferred to 37 °C briefly to decrease the viscosity of the gelatin, after which it was aspirated from the wells and replaced with fresh cell culture media. 

### 2.5. Viability and Proliferation Analysis

Viability of HepG2 and Caco2 organoids following immersion bioprinting in both the Inkredible and Allevi2 bioprinters was assessed on days 1, 3, 5, and 7 (n = 3) using a standard live/dead cell viability kit (Thermo Fisher, Waltham, MA, USA) according to the manufacturer’s recommendations. Stained organoids were imaged using macro-confocal microscopy (Leica TCS LSI, Leica Microsystems, Buffalo Grove, IL, USA) to visualize viable cells (green) and dead cells (red). This study was also applied to A549 lung epithelial cell-based organoids, of which the cells were cultured in the same manner as the other cell lines.

Proliferation of cells within the different organoids was evaluated on days 1, 3, 5, and 7 (n = 3) by quantification of mitochondrial metabolism with a CellTiter 96^®^ Aqueous One Solution Cell Proliferation Assay kit (Promega, Madison, WI, USA) according to the manufacturer’s protocol. Absorbance was quantified on a Varioskan Lux multimode plate reader (Thermo Fisher) at 490 nm. Cell number was proportional to the absorbance signal.

### 2.6. Patient-Derived Tumor Biospecimen Processing

Two glioblastoma (GBM) biospecimens and one sarcoma biospecimen were obtained from 3 surgically treated patients in adherence to the guidelines of the Wake Forest Baptist Medical Center IRB protocols. All biospecimens were completely de-identified prior to use. The specimens were placed in RPMI and transferred fresh to the laboratory by a dedicated tissue procurement manager. Clinical information was not shared with the lab with the exception of the type of tumor and type of prior treatments (if any). Once received, biospecimens were washed in phosphate-buffered saline (PBS) with 2% penicillin-streptomycin for three 5 min cycles. Tissues were individually minced and placed into serum-free low glucose DMEM with 2% penicillin-streptomycin and collagenase (Vitacyte, Indianapolis, IN) and protease (Vitacyte) for 2 h on a shaker plate in 37 °C. Media for glioblastoma biospecimens was also supplemented with 3.3 mg/mL hyaluronidase (STEMCELL Technologies, Seattle, WA, USA). Digested tissues were neutralized with cold serum-supplemented high glucose DMEM and then filtered through a 100 μm cell filter and centrifuged to create a cell pellet. Plasma and non-cellular material were removed, and the pellet was re-suspended in 1 mL BD PharmLyse (BD, San Diego, CA, USA) with 9 mL deionized water for 15 min protected from light. The conical was then centrifuged at 200 rpm for 5 min and the pellet was suspended in media and counted. The cell suspension was then centrifuged to a pellet and then suspended in PBS along with dead cell labeling solution from a dead cell removal kit (Miltenyi Biotec, Germany). The cell suspension was then sorted using a magnetic column to remove the dead cells labeled with microbeads. The effluent cell suspension was centrifuged at 1200 rpm for 5 min and the pellet was suspended in media and counted for use. It should be noted that cell suspensions are not subjected to any cell sorting or isolation protocols so that tumor heterogeneity can be best preserved.

### 2.7. Patient-Derived Tumor Organoid Chemotherapy Screening

GBM 1, GBM 2, and sarcoma PTO sets were prepared in 96-well plates by immersion bioprinting in the same manner as described above for the cell line organoid studies. Bioprinted PTOs were maintained in DMEM-HG (Lonza) supplemented with 10% FBS (Hyclone), 1% L-glutamine (Hyclone) and 1% penicillin/streptomycin (P/S; Hyclone) at 37 °C with 5% CO_2_.

Proof-of-principle drug screening experiments were initiated on day 7 following PTO immersion bioprinting and involved administering the drug compounds to the organoids and incubating under these conditions for 72 h. For GBM PTOs, drug response was assessed for dacomitinib (Selleckchem, Houston, TX, USA) at 6, 600 M, and 60 μM concentrations and an experimental p53 pathway activator (NSC59984, Selleckchem) at 1 μM, 10 μM, and 100 μM concentrations. For sarcoma PTOs, drug response was assessed for imatinib (Selleckchem) at 5 μM, 50 μM, and 500 μM concentrations and doxorubicin (Sigma Aldrich) at 10, 1, and 100 μM concentrations.

Following the 72-h incubation of PTOs with drug compounds, relative viability was assessed by quantifying adenosine triphosphate (ATP) activity using Celltiter Glo 3D assays and measuring relative luminescence using a Varioskan Lux multimode plate reader (Thermo Fisher). Cell number was proportional to the luminescence signal.

## 3. Results and Discussion

### 3.1. Hydrogel Bioink Formulation Immersion Bioprinting Evaluation

First, we tested the capability of the two bioprinters to print correctly according to the gcode that was generated for 96-well plate printing. This was performed by simply printing volumes of water onto the lid of 96-well plates to assess whether or not the printed material was deposited in the centers of the wells. As can be seen in Figure S1, initially the Cellink Inkredible had superior print accuracy, depositing fluid in the center of each well location. In contrast, the Allevi Allevi2 suffered from a drift during the printing procedure resulting in fluid depositions that ended up off-center in the well locations. Upon identification, this problem fortunately was corrected with a software upgrade from the manufacturer, thus allowing us to continue using both bioprinters in parallel for several of the subsequent studies. 

**Figure 1:**
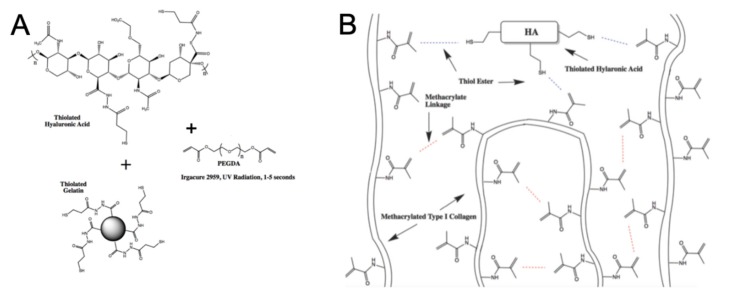
Hydrogel compositions. (**A**) The HyStem hydrogel system, comprised of thiolated hyaluronic acid, thiolated gelatin, and a polyethylene glycol diacrylate (PEGDA) crosslinker. (**B**) Our collagen–hyaluronic acid (HA) hydrogel formulation, comprised of methacrylated collagen and thiolated hyaluronic acid.

**Figure 2:**
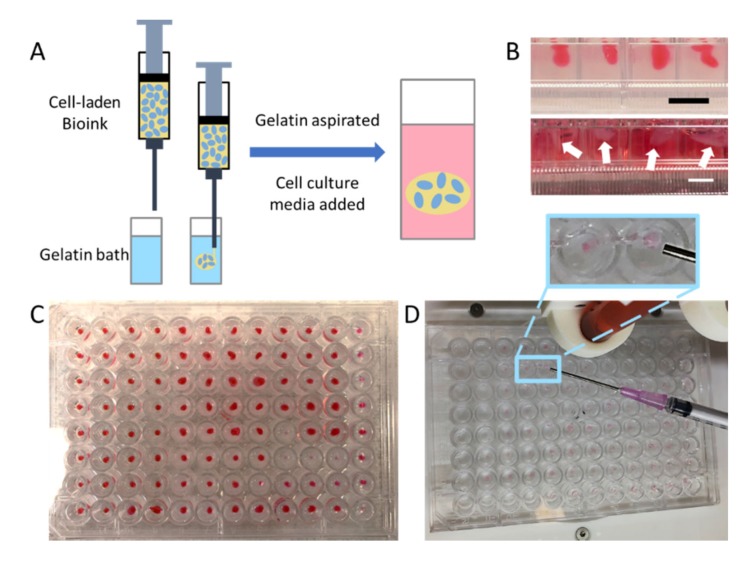
Schematic of the printing process using 2 bioinks in two commercially available bioprinters. The CellInk Inkredible and Allevi 2 printers were used. (**A**) Diagram of immersion printing into a support gelatin bath. Once printing is completed, the support gelatin bath is removed and the cells are left to culture in media. (**B**) Images of the organoids in the 96-well plate are shown at both steps of the process (scale bar = 5 mm). (**C**) Image of thiolated hyaluronic acid (HA) and methacrylated collagen immersed in gelatin with Irgacure (crosslinking agent). (**D**) Image of Hystem hydrogel. Inset image: With the HyStem hydrogel, we ran into problems with it crosslinking mid-print, thereby connecting wells with hydrogel.

With both bioprinting platforms functional, the hydrogels were employed in test experiments. The HyStem–HP hydrogel (Figure 1A) and the collagen–HA hydrogel (Figure 1B) were tested without cells for use in immersion bioprinting protocols. To do this, the hydrogels were deposited in approximately 20 μL volumes within the 10 mg/mL gelatin solutions that had been added to each well of 96-well plates Figure 2A,B. Using default printer hardware settings, the Inkredible bioprinter printed an entire 96-well plate in 8 min, compared to the Allevi2 bioprinter which took 19 min. It should be noted that with newer bioprinter hardware platforms or updated components such as the motors that drive XYZ axes control, these print times could likely be significantly shortened further. More importantly, these test prints revealed a crucial difference between the two hydrogel formulations. The collagen–HA hydrogel performed quite well and was able to be deposited in discrete volumes into the gelatin immersion baths (Figure 2C). Moreover, provided that we included the photoinitiator agent also in the gelatin immersion bath, we could successfully induce a secondary crosslinking reaction between remaining methacrylate groups to further stabilize the printed constructs. The HyStem hydrogel was more difficult to bioprint. While it could be extruded quite easily, the material was undergoing spontaneous pH-driven thiol-acrylate crosslinking, and as a result, filaments of hydrogel do not disconnect from the printed constructs and form connected strands from well to well (Figure 2D). As such, we moved forward with the collagen–HA bioink for subsequent studies.

These results underscore the importance of considering crosslinking methods and kinetics, as well as the mechanical properties of a bioink. HyStem, in its native out-of-the-box formulation, fails as an easy to use bioink due to its spontaneous crosslinking upon mixing together its three components. We have addressed this limitation in previous efforts in which we employed the thiolated HA and gelatin components, but altered the crosslinking method in a number of ways to either change the gelation kinetics [29] or to imbue multiple crosslinking steps to generate partially crosslinked hydrogels that can be extruded and further crosslinked following printing [27,28,30]. These approaches were successful in transitioning HyStem to a more bioprinter-friendly material. In recent years, we have also understood the importance of the presence of Type I collagen in ECM-based biomaterials [33]. Type I collagen allows many cell types to reorganize their physical microenvironment by bundling collagen fibrils into more substantial fiber structures, thus generating in vivo-like collagen architectures [39]. These fiber structures and architectures play a significant role in biology, both in healthy tissue generation and function and in disease states in which ECM composition and architecture changes over time. This understanding has provided the motivation to include Type I collagen in our evolving bioink technologies, despite being more difficult to work with than other components such as gelatin. 

These studies also illustrated the differences in consistency and speed at which these organoid constructs could be formed by different approaches. Organoids could easily be created by hand with a single- or multi-channel pipette. However, placement of the bioink in the same locations in each well would likely be much less consistent. Liquid handling devices could be potentially employed but might have trouble with the viscous mechanical properties of a bioink. Our main motivation for using bioprinting in this setting is in the case of using such patient-derived organoids in a diagnostic application—screening a variety of drug compounds across concentration ranges to identify therapeutic options and or combinations that could be used to better treat actual patients. In such a scenario, a large number of organoids would be necessary, and consistency would be critical. Hence why an automated biofabrication method designed to handle the mechanical properties of a 3D structure-supporting bioink would be attractive compared to hand deposition or by a liquid handler.

**Figure 3:**
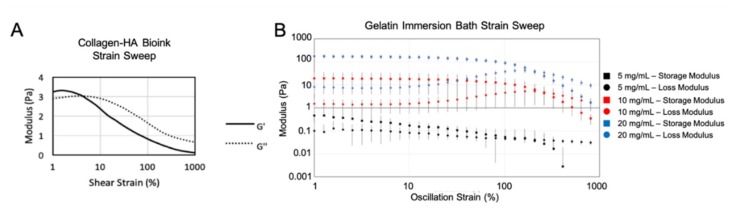
Rheological assessment of collagen–HA bioink versus gelatin bath solution. Storage and loss moduli data resulting from strain sweep tests from 1% to 1000% shear strain (γ) for (**A**) the collagen–HA bioink and (**B**) gelatin solutions (5, 10, and 20 mg/mL).

For successful immersion bioprinting, the bioink itself is but one of two important biomaterial factors; the other is the gelatin immersion bath. Through rheological testing and print tests using the collagen–HA bioink, it was determined that if the gelatin concentration was too great, the storage modulus of the bath would be too high, preventing formation of a spherical organoid. The overly stiff bath would compress upon the extruded bioink and push it out of the bath along the channel created by the immersed nozzle. If the gelatin concentration was too low, the storage modulus would be too low, causing the bioink to not be confined to a spherical shape and diffuse through the loose bath. To maintain a roughly spherical shape after deposition until crosslinked into a stable construct, the storage modulus of the gelatin bath must be a similar magnitude of the storage modulus of the bioink. As shown in Figure 3A, the bioink storage modulus was between 3 and 4 Pa at low strain. The gelatin formulation that worked the best with this bioink was 10 mg/mL, which was approximately 10 Pa at low strain (Figure 3B)—within an order of magnitude of the bioink. The 20 mg/mL solution is too stiff of a solid as shown by the magnitude of, and the difference between, *G*′ and *G*″. The 5 mg/mL solution was a liquid and thus was too loose for this application. The 10 mg/mL was a soft solid that produced the most consistent and optimal printing results.

Other approaches have been developed to also address these limitations, including the use of superhydrophobic surfaces or non-cell adherent surfaces [40,41]. These may very well also be feasible approaches to the problem of bioprinting large numbers of 3D constructs. Our lab has also employed the use of thin polydimethylsiloxane (PDMS) coatings in well plates to generate hydrophobic surfaces to discourage hydrogel constructs from spreading and contacting well walls, it is difficult, even with a consistent bioprinter to ensure such outcomes. As such, as a 3D biomaterials-based laboratory, we employed a 3D approach to address the problem.

### 3.2. Cell Line Tumor Organoid Bioprinting and Viability/Proliferation Analysis

**Figure 4:**
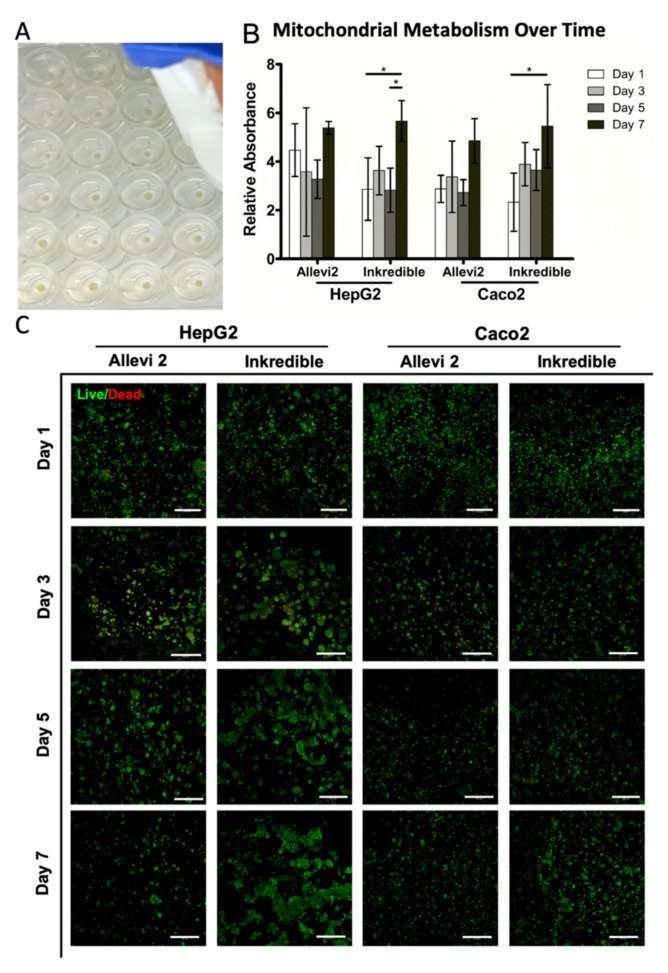
Metabolic activity and viability over time of organoids formed using HepG2 and Caco2 cells. (**A**) A representative image of more fine-tuned organoids (supplemented with gelatin) for visualization, in 96-well plates with greater consistency of geometry and size. (**B**) Quantification of mitochondrial metabolism using (3-(4,5-dimethylthiazol-2-yl)-5-(3-carboxymethoxyphenyl)-2-(4-sulfophenyl)-2H-tetrazolium) (MTS) assays. The higher the metabolic activity, the higher the quantity of cells. Significance: * *p* < 0.05. (**C**) Live/dead staining of organoids over time, showing a high prevalence of viable cells. Green—calcein AM-stained viable cells; red—ethidium homodimer-1-stained dead cells. Scale bar = 150 μm.

Following the establishment of all printing parameters, we next sought to employ the immersion bioprinting approach with cells to evaluate whether or not the printing process itself or the biomaterial characteristics had any detrimental effects on the cells. At this point, the bioprinting process had been fine-tuned resulting in more consistent organoids both in terms of geometry and size, as shown in a representative image in Figure 4A. For this, we chose two relatively common cell lines, HepG2 human hepatoma cells and Caco2 human colorectal cancer epithelial cells, that are often used for biocompatibility studies. Although both cell lines are tumorigenic in origin, they are of relatively low malignancy and are often employed as model cells in place of hepatocytes and gut epithelial cells, respectively. Organoid immersion bioprinting of both cell lines was performed using both bioprinters. To evaluate viability and proliferation, two assays were used. MTS (3-(4,5-dimethylthiazol-2-yl)-5-(3-carboxymethoxyphenyl)-2-(4-sulfophenyl)-2H-tetrazolium) assays were used to measure relative total mitochondrial metabolism, which is proportional to cell number, on day 1, day 3, day 5, and day 7 following bioprinting. For each cell line and printer combination, we see absorbance values that generally increase with time (Figure 4B), indicating successful cell proliferation in the organoid constructs. In addition to MTS assays, live/dead staining and subsequent fluorescent microscopy were used to visualize viable versus dead cells on day 1, day 3, day 5, and day 7. For each cell line and printer combination, we observed some dead cells at various time points, but overwhelmingly the vast majority of cells were clearly viable (Figure 4C). In addition, an earlier panel of organoids printed with the Cellink Inkredible bioprinter was also assessed, which also included A549 lung epithelial cells, which also showed high viability (Figure S2). Taken together, the MTS assays and live/dead staining data suggest that neither the bioink, transient presence of the gelatin bath, nor the immersion printing method have any substantial detrimental effects upon the cells making up the organoids. Rather, viability was high, and cells generally proliferated over time. 

### 3.3. Patient-Derived Tumor Organoid Bioprinting and Chemotherapy Screening

**Figure 5:**
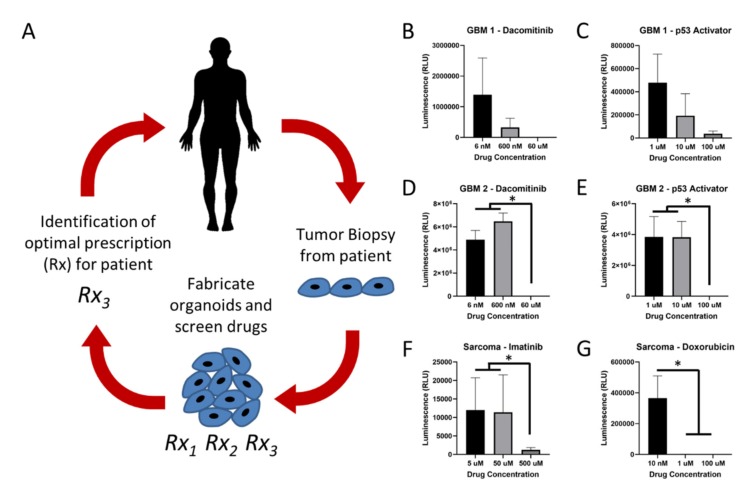
Employing bioprinted patient-derived tumor organoids in a clinical precision medicine setting. (**A**) Tumor tissue is removed from a patient as a biopsy or during surgery. The biospecimen is then dissociated and biofabricated into sets of small organoids or tumor constructs using hydrogel bioinks, after which empirical drug response data can be generated during chemotherapy screening studies. Resulting drug response data can be used to help predict which drugs might be most effective for that specific patient. (**B**–**G**) ATP quantification representing cell viability following chemotherapy screens in immersion bioprinted glioblastoma and sarcoma patient-derived tumor organoids. (**B**,**C**) Glioblastoma (GBM) 1 biospecimen organoids treated with dacomitinib and a p53 pathway activator. (**D**,**E**) GBM 2 biospecimen organoids treated with dacomitinib and a p53 pathway activator. (**F**,**G**) Sarcoma biospecimen organoids treated with imatinib and doxorubicin. Statistical significance: * *p* < 0.05.

As an important proof of principle, we wanted to demonstrate the utility of this bioprinting method to generate sets of tumor organoids derived from biospecimens from specific patients in the clinic. The motivation for this is illustrated in Figure 5A. Ultimately, we hope that patient-derived tumor organoids (PTOs) can be deployed in in vitro drug screening studies to be used as diagnostic tests of sorts, generating empirical drug response data specific to each patient that can help oncologists choose the best drug for a given tumor. As described above, we have performed such in vitro drug screens with PTOs for a number of tumor types [20,21,22,23,42]. However, being able to scale up, automate, and improve the consistency of these PTOs is a hurdle [43]. 

As a demonstration of using immersion bioprinting to great PTOs for chemotherapy screening, we procured three tumor biospecimens from three different patients in the clinic. These were comprised of two glioblastoma biospecimens and one sarcoma specimen (a fibrosarcoma of the skin, specifically). These biospecimens were processed as described above into cell suspensions. Notably, we had a successful yield from each sample. Specifically, the GBM 1 biospecimen yielded approximately 20 million viable cells following dissociation. The GBM 2 biospecimen yielded approximately 10 million viable cells. Lastly, the sarcoma biospecimen yielded approximately 60 million viable cells. Resulting cell suspensions were specifically not sorted so that tumor heterogeneity could be preserved. The cells were combined with the collagen–HA bioink for deployment in immersion bioprinting using the same approach as for the cell line organoids. After bioprinting, the GBM and sarcoma PTOs were maintained for 7 days in the incubator, after which a chemotherapy screening study was initiated. GBM PTOs were subjected to three concentrations of dacomitinib or three concentrations of a p53 activator, while sarcoma organoids were subjected three concentrations of imatinib or three combinations of doxorubicin. Dacomitinib, also known as Vizimpro, is an irreversible inhibitor of EGFR, a target in GBM tumors [44]. The small molecule p53 activator, NSC59984, is not a clinically approved drug, but targets p53 mutant cells, which many GBM tumor cells are [45]. Imatinib is a multi-target inhibitor of tyrosine kinase, platelet-derived growth factor receptor (PDGFR), and c-kit [46]. Doxorubicin, also known as adriamycin, is a widely used antibiotic anthracycline chemotherapy agent that is used for a variety of tumor types [47]. Following drug administration, PTOs were incubated in the presence of each condition for 72 h, after which ATP quantification was used to quantitatively assess drug response. As can be seen in Figure 5B,C, all PTO sets showed positive drug responses, but trends certainly varied. For example, GBM 1 PTOs showed clear dose-dependent decreases in ATP activity with increasing doses of both dacomitinib (Figure 5B) and the p53 activator (Figure 5C), albeit, the large standard deviations made these results not statistically significant. Interestingly, GBM 2 organoids showed no decrease in ATP activity from 6 nM to 600 nM dacomitinib (Figure 5D) and from 1 μM to 10 μM p53 activator (Figure 5E). However, with the increase to the next tested doses, 60 μM dacomitinib and 100 μM p53 activator, ATP activity decreased to nearly no activity—a very significant response. The sarcoma PTOs showed a similar response trend to imatinib, in which there was little difference between 5 μM and 50 μM, but the increase to 500 μM resulted in a significant decrease in ATP activity (Figure 5F). Doxorubicin, on the other hand, generated a significant reduction in ATP activity from 10 nM to 1 μM, with little difference in increasing to 100 μM, as the ATP activity levels were already nearly zero (Figure 5G). 

This set of drug studies provides proof-of-principle data indicating that the immersion bioprinting methodology described herein seems to be a feasible way to potentially scale up PTO biofabrication for use in drug screening studies, and potentially other end applications. In ongoing studies, we are aiming to perform deeper analyses of these PTOs from a biological perspective. For instance, while the studies described in Figure 5 indicate a viable methodology, we wish to also generate histology and biomarker data that characterize and identify the likely heterogeneous cell populations contained within the PTOs. Furthermore, while we believe that in vitro 3D tissue and tumor models generated using biomimetic ECM-derived materials are superior to traditional 2D cell cultures based on functional tests [14,17,19,38] and currently unpublished evidence that 2D cultures can induce changes in drug resistance, we still need to confirm with genetic sequencing that our PTO models are more proficient at maintaining the genomic profiles of the originating tissue biospecimen through the culture duration and reduce genetic drift over time. Such studies are currently ongoing for PTOs of several tumor types. 

## 4. Conclusions

Organoid and tissue chip technology has advanced incredibly quickly in the last decade to the point where clinical deployment is likely inevitable. However, to be considered realistic for clinical diagnostics and personalized medicine, these technologies need to be incredibly consistent, reliable, scalable, and user-friendly. For organoids with more complexity than simple hanging drop-style spheroids, this has been a challenge. Here, we have posited that bioprinting, and more specifically, immersion bioprinting, can be used to overcome this challenge. With advances in bioprinting hardware, software, functional ECM-derived bioinks, and modifications to printing protocols, bioprinting can be harnessed not only to print larger tissue constructs, but also large numbers of micro-scaled tissue and tumor models for applications such as drug development, diagnostics, and personalized medicine. 

## Supplementary Materials

The following are available online at https://www.mdpi.com/2072-666X/11/2/208/s1. Figure S1: Evaluation of bioprinter accuracy in a 96-well format. Figure S2: An earlier iteration of viability analysis in immersion bioprinted cell line-based organoids. File S1: Example gcode for printing 2 columns worth of organoids in a 96-well plate using the Cellink Inkredible bioprinter. File S2: Example gcode for printing 2 columns worth of organoids in a 96-well plate using the Allevi Allevi2 bioprinter.
