# Supplementary material for: Immersion Bioprinting of Tumor Organoids in Multi-Well Plates for Increasing Chemotherapy Screening Throughput
Source: Micromachines (Basel). 2020 Feb 18;11(2):208. doi: 10.3390/mi11020208 (PMC7074680; doi:10.3390/mi11020208)
Supplement: Supplementary file 1 [file micromachines-11-00208-s001.pdf]

Supplemental Materials

Immersion Bioprinting of Tumor Organoids in Multi-Well Plates for Increasing Chemotherapy Screening Throughput

Erin Maloney, Casey Clark, Hemamylammal Sivakumar, KyungMin Yoo, Julio Aleman, Shiny A. P. Rajan, Steven Forsythe, Andrea Mazzocchi, Adrian W. Laxton, Stephen B. Tatter, Roy E. Strowd, Konstantinos I. Votanopoulos, and Aleksander Skardal

Contents:

Supplemental Figures 1 and 2

Supplemental Code Files 1 and 2

Supplemental Figures:

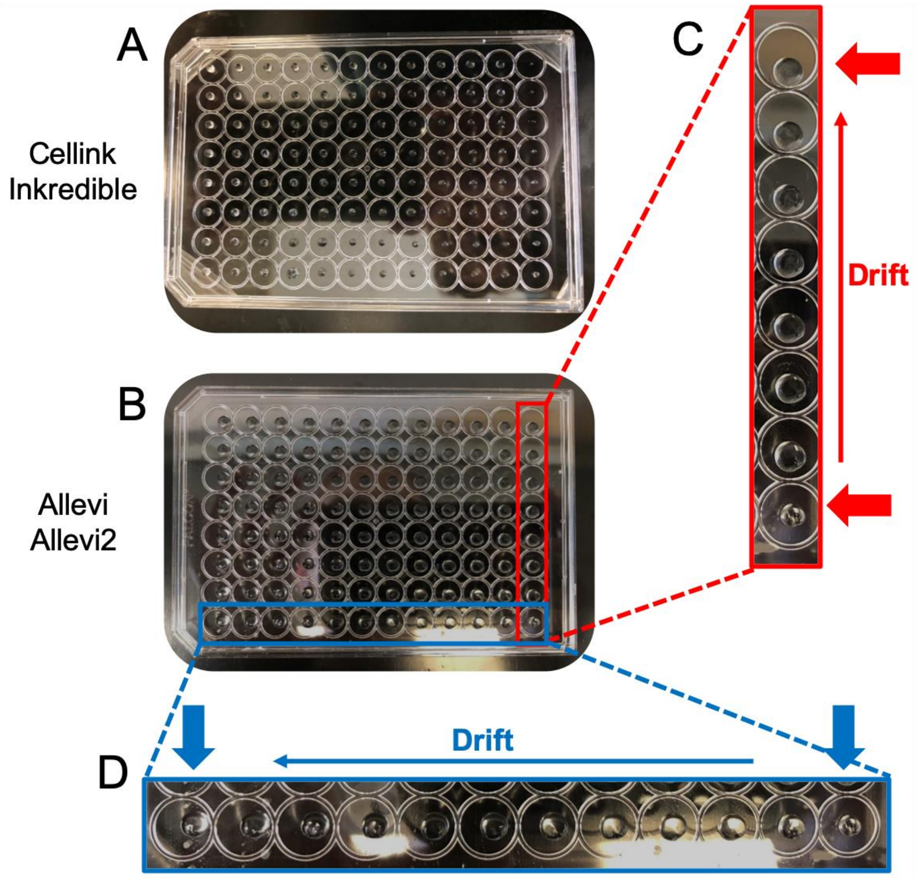

Figure S1. Evaluation of bioprinter accuracy in a 96-well format. The Cellink Inkredible and Allevi2 bioprinters were evaluated for accuracy at printing within the feature of the lid of a 96-well plate. (**A**) The Inkredible generally printed material in the middle of each well location with few problems. (**B**) The Allevi2 initially suffered from printing drift, resulting in depositions that gradually became more off-center as highlighted in the (**C**,**D**) inset images. This drift problem was eventually corrected through a software upgrade from the manufacturer.

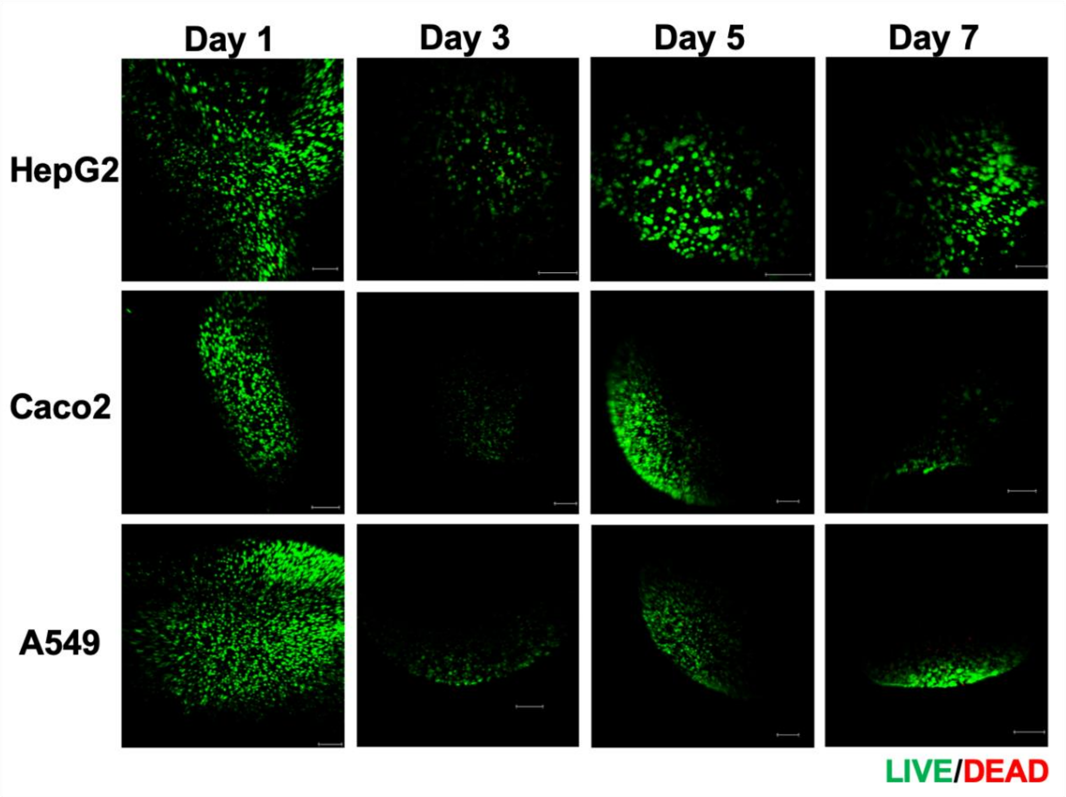

Figure S2. An earlier iteration of viability analysis in immersion bioprinted cell line-based organoids. Live/Dead staining over time of organoids prepared with HepG2 hepatoma cells, Caco2 colorectal cancer epithelial cells, and A549 lung epithelial cells. Green – Calcein AM-stained viable cells; Red – Ethidium homodimer-1-stained dead cells. Scale bar = 150 μm.

Supplemental Code Files:

Supplementary Code File 1 – Example gcode for printing 2 columns worth of organoids in a 96-well plate using the Cellink Inkredible bioprinter.

```
; Post processed by INKREDIBLE post processor, This is the dev branch
; generated by Slic3r 1.2.9 on 2018-06-26 at 10:34:26
; external perimeters extrusion width = 0.40mm
; perimeters extrusion width = 0.42mm
; infill extrusion width = 0.42mm
; solid infill extrusion width = 0.42mm
; top infill extrusion width = 0.42mm
; support material extrusion width = 0.40mm
M104 S200; set temperature
M760
M109 S200; wait for temperature to be reached
G21; set units to millimeters
G90; use absolute coordinates
M83; use relative distances for extrusion
G1 Z0.400 F600.000; move to next layer (0)
M761
```

```
G1 E-2.00000 F0.00000 ; retract
G1 Z2.400 F600.000 ; lift Z
G1 X-2.941 Y31.775 F600.000 ; move to first infill point
G1 Z0.400 F600.000 ; restore layer Z
M760
G1 E2.00000 F0.00000 ; unretract
G1 X-2.941 Y32.216 E0.01103 F600.000 ; infill
G1 X-2.500 Y32.531 E0.01351 ; infill
G1 X-2.500 Y31.473 E0.02639 ; infill
G1 X-2.059 Y31.780 E0.01339 ; infill
G1 X-2.059 Y32.216 E0.01090 ; infill
M761
G1 E-2.00000 F0.00000 ; retract
G1 Z2.400 F600.000 ; lift Z
G1 X-2.500 Y31.403 F600.000 ; move to first infill point
G1 X-2.033 Y28.373 F600.000 ; move to first infill point
G1 X-1.957 Y26.696 F600.000 ; move to first infill point
G1 X-2.135 Y26.538 F600.000 ; move to first infill point
G1 X-2.500 Y23.597 F600.000 ; move to first infill point
G1 X-2.059 Y23.216 F600.000 ; move to first infill point
G1 Z0.400 F600.000 ; restore layer Z
M760
G1 E2.00000 F0.00000 ; unretract
G1 X-2.059 Y22.780 E0.01090 F600.000 ; infill
G1 X-2.500 Y22.473 E0.01339 ; infill
G1 X-2.500 Y23.531 E0.02639 ; infill
G1 X-2.941 Y23.216 E0.01351 ; infill
G1 X-2.941 Y22.775 E0.01103 ; infill
M761
G1 E-2.00000 F0.00000 ; retract
G1 Z2.400 F600.000 ; lift Z
G1 X-2.500 Y22.403 F600.000 ; move to first infill point
G1 X-2.033 Y19.373 F600.000 ; move to first infill point
G1 X-1.957 Y17.696 F600.000 ; move to first infill point
G1 X-2.135 Y17.538 F600.000 ; move to first infill point
G1 X-2.500 Y14.597 F600.000 ; move to first infill point
G1 X-2.059 Y14.216 F600.000 ; move to first infill point
G1 Z0.400 F600.000 ; restore layer Z
M760
G1 E2.00000 F0.00000 ; unretract
G1 X-2.059 Y13.780 E0.01090 F600.000 ; infill
G1 X-2.500 Y13.473 E0.01339 ; infill
```

```
G1 X-2.500 Y14.531 E0.02639 ; infill
G1 X-2.941 Y14.216 E0.01351 ; infill
G1 X-2.941 Y13.775 E0.01103 ; infill
M761
G1 E-2.00000 F0.00000 ; retract
G1 Z2.400 F600.000 ; lift Z
G1 X-2.500 Y13.403 F600.000 ; move to first infill point
G1 X-2.033 Y10.373 F600.000 ; move to first infill point
G1 X-1.957 Y8.696 F600.000 ; move to first infill point
G1 X-2.135 Y8.538 F600.000 ; move to first infill point
G1 X-2.500 Y5.597 F600.000 ; move to first infill point
G1 X-2.059 Y5.216 F600.000 ; move to first infill point
G1 Z0.400 F600.000 ; restore layer Z
M760
G1 E2.00000 F0.00000 ; unretract
G1 X-2.059 Y4.780 E0.01090 F600.000 ; infill
G1 X-2.500 Y4.473 E0.01339 ; infill
G1 X-2.500 Y5.531 E0.02639 ; infill
G1 X-2.941 Y5.216 E0.01351 ; infill
G1 X-2.941 Y4.775 E0.01103 ; infill
M761
G1 E-2.00000 F0.00000 ; retract
G1 Z2.400 F600.000 ; lift Z
G1 X-2.500 Y4.403 F600.000 ; move to first infill point
G1 X-2.033 Y1.373 F600.000 ; move to first infill point
G1 X-1.957 Y-0.304 F600.000 ; move to first infill point
G1 X-2.135 Y-0.462 F600.000 ; move to first infill point
G1 X-2.500 Y-3.403 F600.000 ; move to first infill point
G1 X-2.059 Y-3.784 F600.000 ; move to first infill point
G1 Z0.400 F600.000 ; restore layer Z
M760
G1 E2.00000 F0.00000 ; unretract
G1 X-2.059 Y-4.220 E0.01090 F600.000 ; infill
G1 X-2.500 Y-4.527 E0.01339 ; infill
G1 X-2.500 Y-3.469 E0.02639 ; infill
G1 X-2.941 Y-3.784 E0.01351 ; infill
G1 X-2.941 Y-4.225 E0.01103 ; infill
M761
G1 E-2.00000 F0.00000 ; retract
G1 Z2.400 F600.000 ; lift Z
G1 X-2.500 Y-4.597 F600.000 ; move to first infill point
G1 X-2.033 Y-7.627 F600.000 ; move to first infill point
```

```
G1 X-1.957 Y-9.304 F600.000 ; move to first infill point
G1 X-2.135 Y-9.462 F600.000 ; move to first infill point
G1 X-2.500 Y-12.403 F600.000 ; move to first infill point
G1 X-2.059 Y-12.784 F600.000 ; move to first infill point
G1 Z0.400 F600.000 ; restore layer Z
M760
G1 E2.00000 F0.00000 ; unretract
G1 X-2.059 Y-13.220 E0.01090 F600.000 ; infill
G1 X-2.500 Y-13.527 E0.01339 ; infill
G1 X-2.500 Y-12.469 E0.02639 ; infill
G1 X-2.941 Y-12.784 E0.01351 ; infill
G1 X-2.941 Y-13.225 E0.01103 ; infill
M761
G1 E-2.00000 F0.00000 ; retract
G1 Z2.400 F600.000 ; lift Z
G1 X-2.500 Y-13.597 F600.000 ; move to first infill point
G1 X-2.033 Y-16.627 F600.000 ; move to first infill point
G1 X-1.957 Y-18.304 F600.000 ; move to first infill point
G1 X-2.135 Y-18.462 F600.000 ; move to first infill point
G1 X-2.500 Y-21.403 F600.000 ; move to first infill point
G1 X-2.059 Y-21.784 F600.000 ; move to first infill point
G1 Z0.400 F600.000 ; restore layer Z
M760
G1 E2.00000 F0.00000 ; unretract
G1 X-2.059 Y-22.220 E0.01090 F600.000 ; infill
G1 X-2.500 Y-22.527 E0.01339 ; infill
G1 X-2.500 Y-21.469 E0.02639 ; infill
G1 X-2.941 Y-21.784 E0.01351 ; infill
G1 X-2.941 Y-22.225 E0.01102 ; infill
M761
G1 E-2.00000 F0.00000 ; retract
G1 Z2.400 F600.000 ; lift Z
G1 X-1.901 Y-22.032 F600.000 ; move to first infill point
G1 X2.000 Y-21.848 F600.000 ; move to first infill point
G1 X2.000 Y-21.848 F600.000 ; move to first infill point
G1 X5.901 Y-22.032 F600.000 ; move to first infill point
G1 X6.059 Y-22.225 F600.000 ; move to first infill point
G1 Z0.400 F600.000 ; restore layer Z
M760
G1 E2.00000 F0.00000 ; unretract
G1 X6.059 Y-21.784 E0.01102 F600.000 ; infill
G1 X6.500 Y-21.469 E0.01352 ; infill
```

```
G1 X6.500 Y-22.527 E0.02639 ; infill
G1 X6.941 Y-22.220 E0.01339 ; infill
G1 X6.941 Y-21.784 E0.01090 ; infill
M761
G1 E-2.00000 F0.00000 ; retract
G1 Z2.400 F600.000 ; lift Z
G1 X6.500 Y-21.403 F600.000 ; move to first infill point
G1 X5.957 Y-18.304 F600.000 ; move to first infill point
G1 X5.907 Y-16.737 F600.000 ; move to first infill point
G1 X6.088 Y-16.579 F600.000 ; move to first infill point
G1 X6.500 Y-13.597 F600.000 ; move to first infill point
G1 X6.059 Y-13.225 F600.000 ; move to first infill point
G1 Z0.400 F600.000 ; restore layer Z
M760
G1 E2.00000 F0.00000 ; unretract
G1 X6.059 Y-12.784 E0.01103 F600.000 ; infill
G1 X6.500 Y-12.469 E0.01352 ; infill
G1 X6.500 Y-13.527 E0.02639 ; infill
G1 X6.941 Y-13.220 E0.01339 ; infill
G1 X6.941 Y-12.784 E0.01090 ; infill
M761
G1 E-2.00000 F0.00000 ; retract
G1 Z2.400 F600.000 ; lift Z
G1 X6.500 Y-12.403 F600.000 ; move to first infill point
G1 X5.957 Y-9.304 F600.000 ; move to first infill point
G1 X5.907 Y-7.737 F600.000 ; move to first infill point
G1 X6.088 Y-7.579 F600.000 ; move to first infill point
G1 X6.500 Y-4.597 F600.000 ; move to first infill point
G1 X6.059 Y-4.225 F600.000 ; move to first infill point
G1 Z0.400 F600.000 ; restore layer Z
M760
G1 E2.00000 F0.00000 ; unretract
G1 X6.059 Y-3.784 E0.01103 F600.000 ; infill
G1 X6.500 Y-3.469 E0.01352 ; infill
G1 X6.500 Y-4.527 E0.02639 ; infill
G1 X6.941 Y-4.220 E0.01339 ; infill
G1 X6.941 Y-3.784 E0.01090 ; infill
M761
G1 E-2.00000 F0.00000 ; retract
G1 Z2.400 F600.000 ; lift Z
G1 X6.500 Y-3.403 F600.000 ; move to first infill point
G1 X5.957 Y-0.304 F600.000 ; move to first infill point
```

```
G1 X5.907 Y1.263 F600.000 ; move to first infill point
G1 X6.088 Y1.421 F600.000 ; move to first infill point
G1 X6.500 Y4.403 F600.000 ; move to first infill point
G1 X6.059 Y4.775 F600.000 ; move to first infill point
G1 Z0.400 F600.000 ; restore layer Z
M760
G1 E2.00000 F0.00000 ; unretract
G1 X6.059 Y5.216 E0.01103 F600.000 ; infill
G1 X6.500 Y5.531 E0.01352 ; infill
G1 X6.500 Y4.473 E0.02639 ; infill
G1 X6.941 Y4.780 E0.01339 ; infill
G1 X6.941 Y5.216 E0.01090 ; infill
M761
G1 E-2.00000 F0.00000 ; retract
G1 Z2.400 F600.000 ; lift Z
G1 X6.500 Y5.597 F600.000 ; move to first infill point
G1 X5.957 Y8.696 F600.000 ; move to first infill point
G1 X5.907 Y10.263 F600.000 ; move to first infill point
G1 X6.088 Y10.421 F600.000 ; move to first infill point
G1 X6.500 Y13.403 F600.000 ; move to first infill point
G1 X6.059 Y13.775 F600.000 ; move to first infill point
G1 Z0.400 F600.000 ; restore layer Z
M760
G1 E2.00000 F0.00000 ; unretract
G1 X6.059 Y14.216 E0.01103 F600.000 ; infill
G1 X6.500 Y14.531 E0.01352 ; infill
G1 X6.500 Y13.473 E0.02639 ; infill
G1 X6.941 Y13.780 E0.01339 ; infill
G1 X6.941 Y14.216 E0.01090 ; infill
M761
G1 E-2.00000 F0.00000 ; retract
G1 Z2.400 F600.000 ; lift Z
G1 X6.500 Y14.597 F600.000 ; move to first infill point
G1 X5.957 Y17.696 F600.000 ; move to first infill point
G1 X5.907 Y19.263 F600.000 ; move to first infill point
G1 X6.088 Y19.421 F600.000 ; move to first infill point
G1 X6.500 Y22.403 F600.000 ; move to first infill point
G1 X6.059 Y22.775 F600.000 ; move to first infill point
G1 Z0.400 F600.000 ; restore layer Z
M760
G1 E2.00000 F0.00000 ; unretract
G1 X6.059 Y23.216 E0.01103 F600.000 ; infill
```

```
G1 X6.500 Y23.531 E0.01352 ; infill
G1 X6.500 Y22.473 E0.02639 ; infill
G1 X6.941 Y22.780 E0.01339 ; infill
G1 X6.941 Y23.216 E0.01090 ; infill
M761
G1 E-2.00000 F0.00000 ; retract
G1 Z2.400 F600.000 ; lift Z
G1 X6.500 Y23.597 F600.000 ; move to first infill point
G1 X5.957 Y26.696 F600.000 ; move to first infill point
G1 X5.907 Y28.263 F600.000 ; move to first infill point
G1 X6.088 Y28.421 F600.000 ; move to first infill point
G1 X6.500 Y31.403 F600.000 ; move to first infill point
G1 X6.059 Y31.775 F600.000 ; move to first infill point
G1 Z0.400 F600.000 ; restore layer Z
M760
G1 E2.00000 F0.00000 ; unretract
G1 X6.059 Y32.216 E0.01103 F600.000 ; infill
G1 X6.500 Y32.531 E0.01352 ; infill
G1 X6.500 Y31.473 E0.02639 ; infill
G1 X6.941 Y31.780 E0.01339 ; infill
G1 X6.941 Y32.216 E0.01090 ; infill
M761
G1 E-2.00000 F0.00000 ; retract
G1 Z2.400 F600.000 ; lift Z
G1 X6.500 Y31.403 F600.000 ; move to first infill point
G1 X2.000 Y2.881 F600.000 ; move to first infill point
G1 X2.000 Y-13.585 F600.000 ; move to first infill point
G1 X2.000 Y-21.848 F600.000 ; move to first infill point
G1 X2.087 Y-26.498 F600.000 ; move to first infill point
G1 X4.512 Y-26.498 F600.000 ; move to first infill point
G1 X4.927 Y-26.499 F600.000 ; move to first infill point
G1 X6.135 Y-27.462 F600.000 ; move to first infill point
G1 X6.500 Y-30.403 F600.000 ; move to first infill point
G1 X6.941 Y-30.784 F600.000 ; move to first infill point
G1 Z0.400 F600.000 ; restore layer Z
M760
G1 E2.00000 F0.00000 ; unretract
G1 X6.941 Y-31.220 E0.01090 F600.000 ; infill
G1 X6.500 Y-31.527 E0.01339 ; infill
G1 X6.500 Y-30.469 E0.02639 ; infill
G1 X6.059 Y-30.784 E0.01352 ; infill
G1 X6.059 Y-31.225 E0.01102 ; infill
```

```
M761
G1 E-2.00000 F0.00000 ; retract
G1 Z2.400 F600.000 ; lift Z
G1 X5.901 Y-31.032 F600.000 ; move to first infill point
G1 X2.047 Y-29.491 F600.000 ; move to first infill point
G1 X1.513 Y-30.078 F600.000 ; move to first infill point
G1 X0.917 Y-30.769 F600.000 ; move to first infill point
G1 X0.787 Y-30.905 F600.000 ; move to first infill point
G1 X-1.901 Y-31.032 F600.000 ; move to first infill point
G1 X-2.059 Y-30.784 F600.000 ; move to first infill point
G1 Z0.400 F600.000 ; restore layer Z
M760
G1 E2.00000 F0.00000 ; unretract
G1 X-2.059 Y-31.220 E0.01090 F600.000 ; infill
G1 X-2.500 Y-31.527 E0.01339 ; infill
G1 X-2.500 Y-30.469 E0.02639 ; infill
G1 X-2.941 Y-30.784 E0.01351 ; infill
G1 X-2.941 Y-31.225 E0.01103 ; infill
G1 Z0.800 F600.000 ; move to next layer (1)
M761
G1 E-2.00000 F0.00000 ; retract
G1 Z2.800 F600.000 ; lift Z
G1 X-2.793 Y31.310 F600.000 ; move to first infill point
G1 Z0.800 F600.000 ; restore layer Z
M760
G1 E2.00000 F0.00000 ; unretract
G1 X-2.207 Y31.310 E0.01088 F600.000 ; infill
G1 X-1.701 Y31.769 E0.01271 ; infill
G1 X-3.299 Y31.769 E0.02971 ; infill
G1 X-3.305 Y32.228 E0.00854 ; infill
G1 X-1.695 Y32.228 E0.02993 ; infill
G1 X-2.206 Y32.688 E0.01277 ; infill
G1 X-2.794 Y32.688 E0.01093 ; infill
M761
G1 E-2.00000 F0.00000 ; retract
G1 Z2.800 F600.000 ; lift Z
G1 X-2.500 Y31.084 F600.000 ; move to first infill point
G1 X-2.046 Y27.744 F600.000 ; move to first infill point
G1 X-2.058 Y27.243 F600.000 ; move to first infill point
G1 X-2.229 Y27.086 F600.000 ; move to first infill point
G1 X-2.500 Y23.913 F600.000 ; move to first infill point
G1 X-2.758 Y23.687 F600.000 ; move to first infill point
```

```
G1 Z0.800 F600.000 ; restore layer Z
M760
G1 E2.00000 F0.00000 ; unretract
G1 X-2.204 Y23.687 E0.01031 F600.000 ; infill
G1 X-1.695 Y23.228 E0.01272 ; infill
G1 X-3.305 Y23.228 E0.02990 ; infill
G1 X-3.299 Y22.769 E0.00852 ; infill
G1 X-1.701 Y22.769 E0.02968 ; infill
G1 X-2.207 Y22.310 E0.01269 ; infill
G1 X-2.793 Y22.310 E0.01087 ; infill
M761
G1 E-2.00000 F0.00000 ; retract
G1 Z2.800 F600.000 ; lift Z
G1 X-2.500 Y22.084 F600.000 ; move to first infill point
G1 X-2.046 Y18.744 F600.000 ; move to first infill point
G1 X-2.058 Y18.243 F600.000 ; move to first infill point
G1 X-2.229 Y18.086 F600.000 ; move to first infill point
G1 X-2.500 Y14.913 F600.000 ; move to first infill point
G1 X-2.758 Y14.687 F600.000 ; move to first infill point
G1 Z0.800 F600.000 ; restore layer Z
M760
G1 E2.00000 F0.00000 ; unretract
G1 X-2.204 Y14.687 E0.01031 F600.000 ; infill
G1 X-1.695 Y14.228 E0.01272 ; infill
G1 X-3.305 Y14.228 E0.02990 ; infill
G1 X-3.299 Y13.769 E0.00852 ; infill
G1 X-1.701 Y13.769 E0.02968 ; infill
G1 X-2.207 Y13.310 E0.01269 ; infill
G1 X-2.793 Y13.310 E0.01087 ; infill
M761
G1 E-2.00000 F0.00000 ; retract
G1 Z2.800 F600.000 ; lift Z
G1 X-2.500 Y13.084 F600.000 ; move to first infill point
G1 X-2.046 Y9.744 F600.000 ; move to first infill point
G1 X-2.058 Y9.243 F600.000 ; move to first infill point
G1 X-2.229 Y9.086 F600.000 ; move to first infill point
G1 X-2.500 Y5.913 F600.000 ; move to first infill point
G1 X-2.758 Y5.687 F600.000 ; move to first infill point
G1 Z0.800 F600.000 ; restore layer Z
M760
G1 E2.00000 F0.00000 ; unretract
G1 X-2.204 Y5.687 E0.01031 F600.000 ; infill
```

```
G1 X-1.695 Y5.228 E0.01272 ; infill
G1 X-3.305 Y5.228 E0.02990 ; infill
G1 X-3.299 Y4.769 E0.00852 ; infill
G1 X-1.701 Y4.769 E0.02968 ; infill
G1 X-2.207 Y4.310 E0.01269 ; infill
G1 X-2.793 Y4.310 E0.01087 ; infill
M761
G1 E-2.00000 F0.00000 ; retract
G1 Z2.800 F600.000 ; lift Z
G1 X-2.500 Y4.084 F600.000 ; move to first infill point
G1 X-2.046 Y0.744 F600.000 ; move to first infill point
G1 X-2.058 Y0.243 F600.000 ; move to first infill point
G1 X-2.229 Y0.086 F600.000 ; move to first infill point
G1 X-2.500 Y-3.087 F600.000 ; move to first infill point
G1 X-2.758 Y-3.313 F600.000 ; move to first infill point
G1 Z0.800 F600.000 ; restore layer Z
M760
G1 E2.00000 F0.00000 ; unretract
G1 X-2.204 Y-3.313 E0.01031 F600.000 ; infill
G1 X-1.695 Y-3.772 E0.01272 ; infill
G1 X-3.305 Y-3.772 E0.02990 ; infill
G1 X-3.299 Y-4.231 E0.00852 ; infill
G1 X-1.701 Y-4.231 E0.02968 ; infill
G1 X-2.207 Y-4.690 E0.01269 ; infill
G1 X-2.793 Y-4.690 E0.01087 ; infill
M761
G1 E-2.00000 F0.00000 ; retract
G1 Z2.800 F600.000 ; lift Z
G1 X-2.500 Y-4.916 F600.000 ; move to first infill point
G1 X-2.046 Y-8.256 F600.000 ; move to first infill point
G1 X-2.058 Y-8.757 F600.000 ; move to first infill point
G1 X-2.229 Y-8.914 F600.000 ; move to first infill point
G1 X-2.500 Y-12.087 F600.000 ; move to first infill point
G1 X-2.758 Y-12.313 F600.000 ; move to first infill point
G1 Z0.800 F600.000 ; restore layer Z
M760
G1 E2.00000 F0.00000 ; unretract
G1 X-2.204 Y-12.313 E0.01031 F600.000 ; infill
G1 X-1.695 Y-12.772 E0.01272 ; infill
G1 X-3.305 Y-12.772 E0.02990 ; infill
G1 X-3.299 Y-13.231 E0.00852 ; infill
G1 X-1.701 Y-13.231 E0.02968 ; infill
```

```
G1 X-2.207 Y-13.690 E0.01269 ; infill
G1 X-2.793 Y-13.690 E0.01087 ; infill
M761
G1 E-2.00000 F0.00000 ; retract
G1 Z2.800 F600.000 ; lift Z
G1 X-2.500 Y-13.916 F600.000 ; move to first infill point
G1 X-2.046 Y-17.256 F600.000 ; move to first infill point
G1 X-2.058 Y-17.757 F600.000 ; move to first infill point
G1 X-2.229 Y-17.914 F600.000 ; move to first infill point
G1 X-2.500 Y-21.087 F600.000 ; move to first infill point
G1 X-2.758 Y-21.313 F600.000 ; move to first infill point
G1 Z0.800 F600.000 ; restore layer Z
M760
G1 E2.00000 F0.00000 ; unretract
G1 X-2.204 Y-21.313 E0.01031 F600.000 ; infill
G1 X-1.695 Y-21.772 E0.01272 ; infill
G1 X-3.305 Y-21.772 E0.02990 ; infill
G1 X-3.299 Y-22.231 E0.00852 ; infill
G1 X-1.701 Y-22.231 E0.02968 ; infill
G1 X-2.207 Y-22.690 E0.01269 ; infill
G1 X-2.793 Y-22.690 E0.01087 ; infill
M761
G1 E-2.00000 F0.00000 ; retract
G1 Z2.800 F600.000 ; lift Z
G1 X-1.598 Y-22.151 F600.000 ; move to first infill point
G1 X2.000 Y-22.567 F600.000 ; move to first infill point
G1 X2.000 Y-22.496 F600.000 ; move to first infill point
G1 X5.590 Y-22.081 F600.000 ; move to first infill point
G1 X6.207 Y-22.690 F600.000 ; move to first infill point
G1 Z0.800 F600.000 ; restore layer Z
M760
G1 E2.00000 F0.00000 ; unretract
G1 X6.793 Y-22.690 E0.01087 F600.000 ; infill
G1 X7.299 Y-22.231 E0.01269 ; infill
G1 X5.701 Y-22.231 E0.02968 ; infill
G1 X5.695 Y-21.772 E0.00852 ; infill
G1 X7.305 Y-21.772 E0.02990 ; infill
G1 X6.796 Y-21.313 E0.01272 ; infill
G1 X6.242 Y-21.313 E0.01031 ; infill
M761
G1 E-2.00000 F0.00000 ; retract
G1 Z2.800 F600.000 ; lift Z
```

```
G1 X6.500 Y-21.087 F600.000 ; move to first infill point
G1 X6.058 Y-17.757 F600.000 ; move to first infill point
G1 X6.046 Y-17.256 F600.000 ; move to first infill point
G1 X6.218 Y-17.099 F600.000 ; move to first infill point
G1 X6.500 Y-13.916 F600.000 ; move to first infill point
G1 X6.207 Y-13.690 F600.000 ; move to first infill point
G1 Z0.800 F600.000 ; restore layer Z
M760
G1 E2.00000 F0.00000 ; unretract
G1 X6.793 Y-13.690 E0.01088 F600.000 ; infill
G1 X7.299 Y-13.231 E0.01271 ; infill
G1 X5.701 Y-13.231 E0.02971 ; infill
G1 X5.695 Y-12.772 E0.00854 ; infill
G1 X7.305 Y-12.772 E0.02993 ; infill
G1 X6.794 Y-12.312 E0.01277 ; infill
G1 X6.206 Y-12.312 E0.01093 ; infill
M761
G1 E-2.00000 F0.00000 ; retract
G1 Z2.800 F600.000 ; lift Z
G1 X6.500 Y-12.087 F600.000 ; move to first infill point
G1 X6.058 Y-8.757 F600.000 ; move to first infill point
G1 X6.046 Y-8.256 F600.000 ; move to first infill point
G1 X6.218 Y-8.099 F600.000 ; move to first infill point
G1 X6.500 Y-4.916 F600.000 ; move to first infill point
G1 X6.207 Y-4.690 F600.000 ; move to first infill point
G1 Z0.800 F600.000 ; restore layer Z
M760
G1 E2.00000 F0.00000 ; unretract
G1 X6.793 Y-4.690 E0.01088 F600.000 ; infill
G1 X7.299 Y-4.231 E0.01271 ; infill
G1 X5.701 Y-4.231 E0.02971 ; infill
G1 X5.695 Y-3.772 E0.00854 ; infill
G1 X7.305 Y-3.772 E0.02993 ; infill
G1 X6.794 Y-3.312 E0.01277 ; infill
G1 X6.206 Y-3.312 E0.01093 ; infill
M761
G1 E-2.00000 F0.00000 ; retract
G1 Z2.800 F600.000 ; lift Z
G1 X6.500 Y-3.087 F600.000 ; move to first infill point
G1 X6.058 Y0.243 F600.000 ; move to first infill point
G1 X6.046 Y0.744 F600.000 ; move to first infill point
G1 X6.218 Y0.901 F600.000 ; move to first infill point
```

```
G1 X6.500 Y4.084 F600.000 ; move to first infill point
G1 X6.207 Y4.310 F600.000 ; move to first infill point
G1 Z0.800 F600.000 ; restore layer Z
M760
G1 E2.00000 F0.00000 ; unretract
G1 X6.793 Y4.310 E0.01088 F600.000 ; infill
G1 X7.299 Y4.769 E0.01271 ; infill
G1 X5.701 Y4.769 E0.02971 ; infill
G1 X5.695 Y5.228 E0.00854 ; infill
G1 X7.305 Y5.228 E0.02993 ; infill
G1 X6.794 Y5.688 E0.01277 ; infill
G1 X6.206 Y5.688 E0.01093 ; infill
M761
G1 E-2.00000 F0.00000 ; retract
G1 Z2.800 F600.000 ; lift Z
G1 X6.500 Y5.913 F600.000 ; move to first infill point
G1 X6.058 Y9.243 F600.000 ; move to first infill point
G1 X6.046 Y9.744 F600.000 ; move to first infill point
G1 X6.218 Y9.901 F600.000 ; move to first infill point
G1 X6.500 Y13.084 F600.000 ; move to first infill point
G1 X6.207 Y13.310 F600.000 ; move to first infill point
G1 Z0.800 F600.000 ; restore layer Z
M760
G1 E2.00000 F0.00000 ; unretract
G1 X6.793 Y13.310 E0.01088 F600.000 ; infill
G1 X7.299 Y13.769 E0.01271 ; infill
G1 X5.701 Y13.769 E0.02971 ; infill
G1 X5.695 Y14.228 E0.00854 ; infill
G1 X7.305 Y14.228 E0.02993 ; infill
G1 X6.794 Y14.688 E0.01277 ; infill
G1 X6.206 Y14.688 E0.01093 ; infill
M761
G1 E-2.00000 F0.00000 ; retract
G1 Z2.800 F600.000 ; lift Z
G1 X6.500 Y14.913 F600.000 ; move to first infill point
G1 X6.058 Y18.243 F600.000 ; move to first infill point
G1 X6.046 Y18.744 F600.000 ; move to first infill point
G1 X6.218 Y18.901 F600.000 ; move to first infill point
G1 X6.500 Y22.084 F600.000 ; move to first infill point
G1 X6.207 Y22.310 F600.000 ; move to first infill point
G1 Z0.800 F600.000 ; restore layer Z
M760
```

```
G1 E2.00000 F0.00000 ; unretract
G1 X6.793 Y22.310 E0.01088 F600.000 ; infill
G1 X7.299 Y22.769 E0.01271 ; infill
G1 X5.701 Y22.769 E0.02971 ; infill
G1 X5.695 Y23.228 E0.00854 ; infill
G1 X7.305 Y23.228 E0.02993 ; infill
G1 X6.794 Y23.688 E0.01277 ; infill
G1 X6.206 Y23.688 E0.01093 ; infill
M761
G1 E-2.00000 F0.00000 ; retract
G1 Z2.800 F600.000 ; lift Z
G1 X6.500 Y23.913 F600.000 ; move to first infill point
G1 X6.058 Y27.243 F600.000 ; move to first infill point
G1 X6.046 Y27.744 F600.000 ; move to first infill point
G1 X6.218 Y27.901 F600.000 ; move to first infill point
G1 X6.500 Y31.084 F600.000 ; move to first infill point
G1 X6.207 Y31.310 F600.000 ; move to first infill point
G1 Z0.800 F600.000 ; restore layer Z
M760
G1 E2.00000 F0.00000 ; unretract
G1 X6.793 Y31.310 E0.01088 F600.000 ; infill
G1 X7.299 Y31.769 E0.01271 ; infill
G1 X5.701 Y31.769 E0.02971 ; infill
G1 X5.695 Y32.228 E0.00854 ; infill
G1 X7.305 Y32.228 E0.02993 ; infill
G1 X6.794 Y32.688 E0.01277 ; infill
G1 X6.206 Y32.688 E0.01093 ; infill
M761
G1 E-2.00000 F0.00000 ; retract
G1 Z2.800 F600.000 ; lift Z
G1 X6.500 Y31.084 F600.000 ; move to first infill point
G1 X2.000 Y1.820 F600.000 ; move to first infill point
G1 X2.000 Y-13.496 F600.000 ; move to first infill point
G1 X2.000 Y-21.653 F600.000 ; move to first infill point
G1 X2.000 Y-24.987 F600.000 ; move to first infill point
G1 X3.510 Y-26.499 F600.000 ; move to first infill point
G1 X5.298 Y-26.501 F600.000 ; move to first infill point
G1 X5.769 Y-26.501 F600.000 ; move to first infill point
G1 X6.229 Y-26.914 F600.000 ; move to first infill point
G1 X6.500 Y-30.087 F600.000 ; move to first infill point
G1 X6.242 Y-30.313 F600.000 ; move to first infill point
G1 Z0.800 F600.000 ; restore layer Z
```

```
M760
G1 E2.00000 F0.00000 ; unretract
G1 X6.796 Y-30.313 E0.01031 F600.000 ; infill
G1 X7.305 Y-30.772 E0.01272 ; infill
G1 X5.695 Y-30.772 E0.02990 ; infill
G1 X5.701 Y-31.231 E0.00852 ; infill
G1 X7.299 Y-31.231 E0.02968 ; infill
G1 X6.793 Y-31.690 E0.01269 ; infill
G1 X6.207 Y-31.690 E0.01087 ; infill
M761
G1 E-2.00000 F0.00000 ; retract
G1 Z2.800 F600.000 ; lift Z
G1 X5.598 Y-31.151 F600.000 ; move to first infill point
G1 X2.082 Y-30.367 F600.000 ; move to first infill point
G1 X1.634 Y-30.684 F600.000 ; move to first infill point
G1 X1.172 Y-31.154 F600.000 ; move to first infill point
G1 X1.106 Y-31.217 F600.000 ; move to first infill point
G1 X-1.590 Y-31.081 F600.000 ; move to first infill point
G1 X-2.793 Y-31.690 F600.000 ; move to first infill point
G1 Z0.800 F600.000 ; restore layer Z
M760
G1 E2.00000 F0.00000 ; unretract
G1 X-2.207 Y-31.690 E0.01087 F600.000 ; infill
G1 X-1.701 Y-31.231 E0.01269 ; infill
G1 X-3.299 Y-31.231 E0.02968 ; infill
G1 X-3.305 Y-30.772 E0.00852 ; infill
G1 X-1.695 Y-30.772 E0.02990 ; infill
G1 X-2.204 Y-30.313 E0.01272 ; infill
G1 X-2.758 Y-30.313 E0.01031 ; infill
G1 Z1.200 F600.000 ; move to next layer (2)
M761
G1 E-2.00000 F0.00000 ; retract
G1 Z3.200 F600.000 ; lift Z
G1 X-1.737 Y32.320 F600.000 ; move to first infill point
G1 Z1.200 F600.000 ; restore layer Z
M760
G1 E2.00000 F0.00000 ; unretract
G1 X-1.737 Y31.650 E0.01567 F600.000 ; infill
G1 X-2.246 Y31.125 E0.01707 ; infill
G1 X-2.246 Y32.875 E0.04089 ; infill
G1 X-2.754 Y32.884 E0.01188 ; infill
G1 X-2.754 Y31.116 E0.04129 ; infill
```

```
G1 X-3.263 Y31.680 E0.01773 ; infill
G1 X-3.263 Y32.350 E0.01567 ; infill
M761
G1 E-2.00000 F0.00000 ; retract
G1 Z3.200 F600.000 ; lift Z
G1 X-2.656 Y31.012 F600.000 ; move to first infill point
G1 X-2.075 Y27.598 F600.000 ; move to first infill point
G1 X-2.245 Y27.245 F600.000 ; move to first infill point
G1 X-2.863 Y26.616 F600.000 ; move to first infill point
G1 X-2.656 Y23.988 F600.000 ; move to first infill point
G1 X-3.263 Y23.350 F600.000 ; move to first infill point
G1 Z1.200 F600.000 ; restore layer Z
M760
G1 E2.00000 F0.00000 ; unretract
G1 X-3.263 Y22.680 E0.01567 F600.000 ; infill
G1 X-2.754 Y22.116 E0.01773 ; infill
G1 X-2.754 Y23.875 E0.04109 ; infill
G1 X-2.246 Y23.875 E0.01188 ; infill
G1 X-2.246 Y22.125 E0.04089 ; infill
G1 X-1.737 Y22.650 E0.01707 ; infill
G1 X-1.737 Y23.320 E0.01567 ; infill
M761
G1 E-2.00000 F0.00000 ; retract
G1 Z3.200 F600.000 ; lift Z
G1 X-2.344 Y22.012 F600.000 ; move to first infill point
G1 X-2.075 Y18.598 F600.000 ; move to first infill point
G1 X-2.075 Y18.402 F600.000 ; move to first infill point
G1 X-2.344 Y14.988 F600.000 ; move to first infill point
G1 X-1.737 Y14.320 F600.000 ; move to first infill point
G1 Z1.200 F600.000 ; restore layer Z
M760
G1 E2.00000 F0.00000 ; unretract
G1 X-1.737 Y13.650 E0.01567 F600.000 ; infill
G1 X-2.246 Y13.125 E0.01707 ; infill
G1 X-2.246 Y14.875 E0.04089 ; infill
G1 X-2.754 Y14.875 E0.01188 ; infill
G1 X-2.754 Y13.125 E0.04089 ; infill
G1 X-3.263 Y13.680 E0.01758 ; infill
G1 X-3.263 Y14.350 E0.01567 ; infill
M761
G1 E-2.00000 F0.00000 ; retract
G1 Z3.200 F600.000 ; lift Z
```

```
G1 X-2.656 Y13.012 F600.000 ; move to first infill point
G1 X-2.075 Y9.598 F600.000 ; move to first infill point
G1 X-2.245 Y9.245 F600.000 ; move to first infill point
G1 X-2.863 Y8.616 F600.000 ; move to first infill point
G1 X-2.656 Y5.988 F600.000 ; move to first infill point
G1 X-3.263 Y5.350 F600.000 ; move to first infill point
G1 Z1.200 F600.000 ; restore layer Z
M760
G1 E2.00000 F0.00000 ; unretract
G1 X-3.263 Y4.680 E0.01567 F600.000 ; infill
G1 X-2.754 Y4.125 E0.01758 ; infill
G1 X-2.754 Y5.875 E0.04089 ; infill
G1 X-2.246 Y5.875 E0.01188 ; infill
G1 X-2.246 Y4.125 E0.04089 ; infill
G1 X-1.737 Y4.650 E0.01707 ; infill
G1 X-1.737 Y5.320 E0.01567 ; infill
M761
G1 E-2.00000 F0.00000 ; retract
G1 Z3.200 F600.000 ; lift Z
G1 X-1.500 Y5.000 F600.000 ; move to first infill point
G1 X2.000 Y5.274 F600.000 ; move to first infill point
G1 X2.000 Y5.274 F600.000 ; move to first infill point
G1 X5.500 Y5.000 F600.000 ; move to first infill point
G1 X5.737 Y5.350 F600.000 ; move to first infill point
G1 Z1.200 F600.000 ; restore layer Z
M760
G1 E2.00000 F0.00000 ; unretract
G1 X5.737 Y4.680 E0.01567 F600.000 ; infill
G1 X6.246 Y4.125 E0.01758 ; infill
G1 X6.246 Y5.875 E0.04089 ; infill
G1 X6.754 Y5.875 E0.01188 ; infill
G1 X6.754 Y4.125 E0.04089 ; infill
G1 X7.263 Y4.650 E0.01707 ; infill
G1 X7.263 Y5.320 E0.01567 ; infill
M761
G1 E-2.00000 F0.00000 ; retract
G1 Z3.200 F600.000 ; lift Z
G1 X6.656 Y5.988 F600.000 ; move to first infill point
G1 X5.968 Y9.500 F600.000 ; move to first infill point
G1 X6.718 Y10.227 F600.000 ; move to first infill point
G1 X6.863 Y10.384 F600.000 ; move to first infill point
G1 X6.656 Y13.012 F600.000 ; move to first infill point
```

```
G1 X7.263 Y14.320 F600.000 ; move to first infill point
G1 Z1.200 F600.000 ; restore layer Z
M760
G1 E2.00000 F0.00000 ; unretract
G1 X7.263 Y13.650 E0.01567 F600.000 ; infill
G1 X6.754 Y13.125 E0.01707 ; infill
G1 X6.754 Y14.875 E0.04089 ; infill
G1 X6.246 Y14.875 E0.01188 ; infill
G1 X6.246 Y13.125 E0.04089 ; infill
G1 X5.737 Y13.680 E0.01758 ; infill
G1 X5.737 Y14.350 E0.01567 ; infill
M761
G1 E-2.00000 F0.00000 ; retract
G1 Z3.200 F600.000 ; lift Z
G1 X6.344 Y14.988 F600.000 ; move to first infill point
G1 X6.075 Y18.402 F600.000 ; move to first infill point
G1 X6.075 Y18.598 F600.000 ; move to first infill point
G1 X6.344 Y22.012 F600.000 ; move to first infill point
G1 X5.737 Y23.350 F600.000 ; move to first infill point
G1 Z1.200 F600.000 ; restore layer Z
M760
G1 E2.00000 F0.00000 ; unretract
G1 X5.737 Y22.680 E0.01567 F600.000 ; infill
G1 X6.246 Y22.116 E0.01773 ; infill
G1 X6.246 Y23.875 E0.04109 ; infill
G1 X6.754 Y23.875 E0.01188 ; infill
G1 X6.754 Y22.125 E0.04089 ; infill
G1 X7.263 Y22.650 E0.01707 ; infill
G1 X7.263 Y23.320 E0.01567 ; infill
M761
G1 E-2.00000 F0.00000 ; retract
G1 Z3.200 F600.000 ; lift Z
G1 X6.656 Y23.988 F600.000 ; move to first infill point
G1 X5.968 Y27.500 F600.000 ; move to first infill point
G1 X6.718 Y28.227 F600.000 ; move to first infill point
G1 X6.863 Y28.384 F600.000 ; move to first infill point
G1 X6.656 Y31.012 F600.000 ; move to first infill point
G1 X7.263 Y32.320 F600.000 ; move to first infill point
G1 Z1.200 F600.000 ; restore layer Z
M760
G1 E2.00000 F0.00000 ; unretract
G1 X7.263 Y31.650 E0.01567 F600.000 ; infill
```

```
G1 X6.754 Y31.125 E0.01707 ; infill
G1 X6.754 Y32.875 E0.04089 ; infill
G1 X6.246 Y32.884 E0.01188 ; infill
G1 X6.246 Y31.116 E0.04129 ; infill
G1 X5.737 Y31.680 E0.01773 ; infill
G1 X5.737 Y32.350 E0.01567 ; infill
M761
G1 E-2.00000 F0.00000 ; retract
G1 Z3.200 F600.000 ; lift Z
G1 X6.500 Y31.000 F600.000 ; move to first infill point
G1 X8.198 Y21.447 F600.000 ; move to first infill point
G1 X8.198 Y15.553 F600.000 ; move to first infill point
G1 X7.659 Y7.592 F600.000 ; move to first infill point
G1 X8.362 Y3.951 F600.000 ; move to first infill point
G1 X7.318 Y1.928 F600.000 ; move to first infill point
G1 X6.245 Y0.755 F600.000 ; move to first infill point
G1 X6.075 Y0.402 F600.000 ; move to first infill point
G1 X6.245 Y0.245 F600.000 ; move to first infill point
G1 X6.500 Y-3.000 F600.000 ; move to first infill point
G1 X5.737 Y-3.650 F600.000 ; move to first infill point
G1 Z1.200 F600.000 ; restore layer Z
M760
G1 E2.00000 F0.00000 ; unretract
G1 X5.737 Y-4.350 E0.01637 F600.000 ; infill
G1 X6.246 Y-4.884 E0.01721 ; infill
G1 X6.246 Y-3.116 E0.04129 ; infill
G1 X6.754 Y-3.116 E0.01188 ; infill
G1 X6.754 Y-4.884 E0.04129 ; infill
G1 X7.263 Y-4.350 E0.01721 ; infill
G1 X7.263 Y-3.680 E0.01567 ; infill
M761
G1 E-2.00000 F0.00000 ; retract
G1 Z3.200 F600.000 ; lift Z
G1 X6.656 Y-4.988 F600.000 ; move to first infill point
G1 X6.075 Y-8.402 F600.000 ; move to first infill point
G1 X6.245 Y-8.755 F600.000 ; move to first infill point
G1 X6.863 Y-9.384 F600.000 ; move to first infill point
G1 X6.656 Y-12.012 F600.000 ; move to first infill point
G1 X7.263 Y-12.680 F600.000 ; move to first infill point
G1 Z1.200 F600.000 ; restore layer Z
M760
G1 E2.00000 F0.00000 ; unretract
```

```
G1 X7.263 Y-13.350 E0.01567 F600.000 ; infill
G1 X6.754 Y-13.884 E0.01721 ; infill
G1 X6.754 Y-12.116 E0.04129 ; infill
G1 X6.246 Y-12.116 E0.01188 ; infill
G1 X6.246 Y-13.884 E0.04129 ; infill
G1 X5.737 Y-13.350 E0.01721 ; infill
G1 X5.737 Y-12.650 E0.01637 ; infill
M761
G1 E-2.00000 F0.00000 ; retract
G1 Z3.200 F600.000 ; lift Z
G1 X6.344 Y-13.988 F600.000 ; move to first infill point
G1 X6.075 Y-17.402 F600.000 ; move to first infill point
G1 X6.075 Y-17.598 F600.000 ; move to first infill point
G1 X6.344 Y-21.012 F600.000 ; move to first infill point
G1 X5.737 Y-21.650 F600.000 ; move to first infill point
G1 Z1.200 F600.000 ; restore layer Z
M760
G1 E2.00000 F0.00000 ; unretract
G1 X5.737 Y-22.320 E0.01567 F600.000 ; infill
G1 X6.246 Y-22.884 E0.01773 ; infill
G1 X6.246 Y-21.116 E0.04129 ; infill
G1 X6.754 Y-21.116 E0.01188 ; infill
G1 X6.754 Y-22.884 E0.04129 ; infill
G1 X7.263 Y-22.350 E0.01721 ; infill
G1 X7.263 Y-21.680 E0.01567 ; infill
M761
G1 E-2.00000 F0.00000 ; retract
G1 Z3.200 F600.000 ; lift Z
G1 X5.512 Y-21.844 F600.000 ; move to first infill point
G1 X2.000 Y-21.569 F600.000 ; move to first infill point
G1 X2.000 Y-21.726 F600.000 ; move to first infill point
G1 X-1.500 Y-22.000 F600.000 ; move to first infill point
G1 X-1.737 Y-21.680 F600.000 ; move to first infill point
G1 Z1.200 F600.000 ; restore layer Z
M760
G1 E2.00000 F0.00000 ; unretract
G1 X-1.737 Y-22.350 E0.01567 F600.000 ; infill
G1 X-2.246 Y-22.884 E0.01721 ; infill
G1 X-2.246 Y-21.116 E0.04129 ; infill
G1 X-2.754 Y-21.116 E0.01188 ; infill
G1 X-2.754 Y-22.884 E0.04129 ; infill
G1 X-3.263 Y-22.320 E0.01773 ; infill
```

```
G1 X-3.263 Y-21.650 E0.01567 ; infill
M761
G1 E-2.00000 F0.00000 ; retract
G1 Z3.200 F600.000 ; lift Z
G1 X-2.656 Y-21.012 F600.000 ; move to first infill point
G1 X-1.968 Y-17.500 F600.000 ; move to first infill point
G1 X-2.245 Y-17.245 F600.000 ; move to first infill point
G1 X-2.863 Y-16.616 F600.000 ; move to first infill point
G1 X-2.656 Y-13.988 F600.000 ; move to first infill point
G1 X-3.263 Y-12.650 F600.000 ; move to first infill point
G1 Z1.200 F600.000 ; restore layer Z
M760
G1 E2.00000 F0.00000 ; unretract
G1 X-3.263 Y-13.350 E0.01637 F600.000 ; infill
G1 X-2.754 Y-13.884 E0.01721 ; infill
G1 X-2.754 Y-12.116 E0.04129 ; infill
G1 X-2.246 Y-12.116 E0.01188 ; infill
G1 X-2.246 Y-13.884 E0.04129 ; infill
G1 X-1.737 Y-13.350 E0.01721 ; infill
G1 X-1.737 Y-12.650 E0.01637 ; infill
M761
G1 E-2.00000 F0.00000 ; retract
G1 Z3.200 F600.000 ; lift Z
G1 X-2.344 Y-12.012 F600.000 ; move to first infill point
G1 X-2.075 Y-8.598 F600.000 ; move to first infill point
G1 X-2.077 Y-8.400 F600.000 ; move to first infill point
G1 X-2.344 Y-4.988 F600.000 ; move to first infill point
G1 X-1.737 Y-3.650 F600.000 ; move to first infill point
G1 Z1.200 F600.000 ; restore layer Z
M760
G1 E2.00000 F0.00000 ; unretract
G1 X-1.737 Y-4.350 E0.01637 F600.000 ; infill
G1 X-2.246 Y-4.884 E0.01721 ; infill
G1 X-2.246 Y-3.116 E0.04129 ; infill
G1 X-2.754 Y-3.116 E0.01188 ; infill
G1 X-2.754 Y-4.884 E0.04129 ; infill
G1 X-3.263 Y-4.350 E0.01721 ; infill
G1 X-3.263 Y-3.650 E0.01637 ; infill
M761
G1 E-2.00000 F0.00000 ; retract
G1 Z3.200 F600.000 ; lift Z
G1 X-2.500 Y-5.000 F600.000 ; move to first infill point
```

```
G1 X-4.461 Y-12.449 F600.000 ; move to first infill point
G1 X-3.916 Y-19.840 F600.000 ; move to first infill point
G1 X-4.317 Y-23.206 F600.000 ; move to first infill point
G1 X-3.318 Y-25.072 F600.000 ; move to first infill point
G1 X-2.077 Y-26.401 F600.000 ; move to first infill point
G1 X-2.075 Y-26.598 F600.000 ; move to first infill point
G1 X-2.245 Y-26.755 F600.000 ; move to first infill point
G1 X-2.500 Y-30.000 F600.000 ; move to first infill point
G1 X-3.263 Y-30.650 F600.000 ; move to first infill point
G1 Z1.200 F600.000 ; restore layer Z
M760
G1 E2.00000 F0.00000 ; unretract
G1 X-3.263 Y-31.350 E0.01637 F600.000 ; infill
G1 X-2.754 Y-31.884 E0.01721 ; infill
G1 X-2.754 Y-30.116 E0.04129 ; infill
G1 X-2.246 Y-30.125 E0.01188 ; infill
G1 X-2.246 Y-31.875 E0.04089 ; infill
G1 X-1.737 Y-31.350 E0.01707 ; infill
G1 X-1.737 Y-30.680 E0.01567 ; infill
M761
G1 E-2.00000 F0.00000 ; retract
G1 Z3.200 F600.000 ; lift Z
G1 X-1.500 Y-31.000 F600.000 ; move to first infill point
G1 X1.901 Y-30.576 F600.000 ; move to first infill point
G1 X2.099 Y-30.576 F600.000 ; move to first infill point
G1 X2.256 Y-30.746 F600.000 ; move to first infill point
G1 X5.500 Y-31.000 F600.000 ; move to first infill point
G1 X5.737 Y-30.650 F600.000 ; move to first infill point
G1 Z1.200 F600.000 ; restore layer Z
M760
G1 E2.00000 F0.00000 ; unretract
G1 X5.737 Y-31.320 E0.01567 F600.000 ; infill
G1 X6.246 Y-31.884 E0.01773 ; infill
G1 X6.246 Y-30.116 E0.04129 ; infill
G1 X6.754 Y-30.116 E0.01188 ; infill
G1 X6.754 Y-31.884 E0.04129 ; infill
G1 X7.263 Y-31.350 E0.01721 ; infill
G1 X7.263 Y-30.680 E0.01567 ; infill
G1 Z1.600 F600.000 ; move to next layer (3)
M761
G1 E-2.00000 F0.00000 ; retract
G1 Z3.600 F600.000 ; lift Z
```

```
G1 X-2.245 Y31.252 F600.000 ; move to first infill point
G1 Z1.600 F600.000 ; restore layer Z
M760
G1 E2.00000 F0.00000 ; unretract
G1 X-2.762 Y31.252 E0.01093 F600.000 ; infill
G1 X-3.268 Y31.626 E0.01328 ; infill
G1 X-1.732 Y31.626 E0.03245 ; infill
G1 X-1.655 Y31.999 E0.00805 ; infill
G1 X-3.344 Y31.999 E0.03568 ; infill
G1 X-3.270 Y32.372 E0.00803 ; infill
G1 X-1.734 Y32.372 E0.03244 ; infill
G1 X-2.234 Y32.745 E0.01318 ; infill
G1 X-2.766 Y32.745 E0.01123 ; infill
M761
G1 E-2.00000 F0.00000 ; retract
G1 Z3.600 F600.000 ; lift Z
G1 X-2.500 Y31.085 F600.000 ; move to first infill point
G1 X-2.062 Y27.759 F600.000 ; move to first infill point
G1 X-2.059 Y27.242 F600.000 ; move to first infill point
G1 X-2.230 Y27.085 F600.000 ; move to first infill point
G1 X-2.500 Y23.913 F600.000 ; move to first infill point
G1 X-2.766 Y23.745 F600.000 ; move to first infill point
G1 Z1.600 F600.000 ; restore layer Z
M760
G1 E2.00000 F0.00000 ; unretract
G1 X-2.234 Y23.745 E0.01123 F600.000 ; infill
G1 X-1.734 Y23.372 E0.01318 ; infill
G1 X-3.270 Y23.372 E0.03244 ; infill
G1 X-3.344 Y22.999 E0.00803 ; infill
G1 X-1.655 Y22.999 E0.03568 ; infill
G1 X-1.732 Y22.626 E0.00805 ; infill
G1 X-3.268 Y22.626 E0.03245 ; infill
G1 X-2.762 Y22.252 E0.01328 ; infill
G1 X-2.245 Y22.252 E0.01093 ; infill
M761
G1 E-2.00000 F0.00000 ; retract
G1 Z3.600 F600.000 ; lift Z
G1 X-2.500 Y22.085 F600.000 ; move to first infill point
G1 X-2.062 Y18.759 F600.000 ; move to first infill point
G1 X-2.059 Y18.242 F600.000 ; move to first infill point
G1 X-2.230 Y18.085 F600.000 ; move to first infill point
G1 X-2.500 Y14.913 F600.000 ; move to first infill point
```

```
G1 X-2.766 Y14.745 F600.000 ; move to first infill point
G1 Z1.600 F600.000 ; restore layer Z
M760
G1 E2.00000 F0.00000 ; unretract
G1 X-2.234 Y14.745 E0.01123 F600.000 ; infill
G1 X-1.734 Y14.372 E0.01318 ; infill
G1 X-3.270 Y14.372 E0.03244 ; infill
G1 X-3.344 Y13.999 E0.00803 ; infill
G1 X-1.655 Y13.999 E0.03568 ; infill
G1 X-1.732 Y13.626 E0.00805 ; infill
G1 X-3.268 Y13.626 E0.03245 ; infill
G1 X-2.762 Y13.252 E0.01328 ; infill
G1 X-2.245 Y13.252 E0.01093 ; infill
M761
G1 E-2.00000 F0.00000 ; retract
G1 Z3.600 F600.000 ; lift Z
G1 X-2.500 Y13.085 F600.000 ; move to first infill point
G1 X-2.062 Y9.759 F600.000 ; move to first infill point
G1 X-2.059 Y9.242 F600.000 ; move to first infill point
G1 X-2.230 Y9.085 F600.000 ; move to first infill point
G1 X-2.500 Y5.913 F600.000 ; move to first infill point
G1 X-2.766 Y5.745 F600.000 ; move to first infill point
G1 Z1.600 F600.000 ; restore layer Z
M760
G1 E2.00000 F0.00000 ; unretract
G1 X-2.234 Y5.745 E0.01123 F600.000 ; infill
G1 X-1.734 Y5.372 E0.01318 ; infill
G1 X-3.270 Y5.372 E0.03244 ; infill
G1 X-3.344 Y4.999 E0.00803 ; infill
G1 X-1.655 Y4.999 E0.03568 ; infill
G1 X-1.732 Y4.626 E0.00805 ; infill
G1 X-3.268 Y4.626 E0.03245 ; infill
G1 X-2.762 Y4.252 E0.01328 ; infill
G1 X-2.245 Y4.252 E0.01093 ; infill
M761
G1 E-2.00000 F0.00000 ; retract
G1 Z3.600 F600.000 ; lift Z
G1 X-1.587 Y4.984 F600.000 ; move to first infill point
G1 X2.000 Y5.109 F600.000 ; move to first infill point
G1 X2.000 Y5.415 F600.000 ; move to first infill point
G1 X5.594 Y5.116 F600.000 ; move to first infill point
G1 X6.234 Y5.745 F600.000 ; move to first infill point
```

```
G1 Z1.600 F600.000 ; restore layer Z
M760
G1 E2.00000 F0.00000 ; unretract
G1 X6.766 Y5.745 E0.01123 F600.000 ; infill
G1 X7.266 Y5.372 E0.01318 ; infill
G1 X5.731 Y5.372 E0.03241 ; infill
G1 X5.655 Y4.999 E0.00804 ; infill
G1 X7.345 Y4.999 E0.03568 ; infill
G1 X7.268 Y4.626 E0.00805 ; infill
G1 X5.732 Y4.626 E0.03245 ; infill
G1 X6.238 Y4.252 E0.01328 ; infill
G1 X6.755 Y4.252 E0.01093 ; infill
M761
G1 E-2.00000 F0.00000 ; retract
G1 Z3.600 F600.000 ; lift Z
G1 X6.500 Y5.913 F600.000 ; move to first infill point
G1 X6.059 Y9.242 F600.000 ; move to first infill point
G1 X6.046 Y9.745 F600.000 ; move to first infill point
G1 X6.218 Y9.902 F600.000 ; move to first infill point
G1 X6.500 Y13.085 F600.000 ; move to first infill point
G1 X6.755 Y13.252 F600.000 ; move to first infill point
G1 Z1.600 F600.000 ; restore layer Z
M760
G1 E2.00000 F0.00000 ; unretract
G1 X6.238 Y13.252 E0.01093 F600.000 ; infill
G1 X5.732 Y13.626 E0.01328 ; infill
G1 X7.268 Y13.626 E0.03245 ; infill
G1 X7.345 Y13.999 E0.00805 ; infill
G1 X5.655 Y13.999 E0.03568 ; infill
G1 X5.731 Y14.372 E0.00804 ; infill
G1 X7.266 Y14.372 E0.03241 ; infill
G1 X6.766 Y14.745 E0.01318 ; infill
G1 X6.234 Y14.745 E0.01123 ; infill
M761
G1 E-2.00000 F0.00000 ; retract
G1 Z3.600 F600.000 ; lift Z
G1 X6.500 Y14.913 F600.000 ; move to first infill point
G1 X6.059 Y18.242 F600.000 ; move to first infill point
G1 X6.046 Y18.745 F600.000 ; move to first infill point
G1 X6.218 Y18.902 F600.000 ; move to first infill point
G1 X6.500 Y22.085 F600.000 ; move to first infill point
G1 X6.755 Y22.252 F600.000 ; move to first infill point
```

```
G1 Z1.600 F600.000 ; restore layer Z
M760
G1 E2.00000 F0.00000 ; unretract
G1 X6.238 Y22.252 E0.01093 F600.000 ; infill
G1 X5.732 Y22.626 E0.01328 ; infill
G1 X7.268 Y22.626 E0.03245 ; infill
G1 X7.345 Y22.999 E0.00805 ; infill
G1 X5.655 Y22.999 E0.03568 ; infill
G1 X5.731 Y23.372 E0.00804 ; infill
G1 X7.266 Y23.372 E0.03241 ; infill
G1 X6.766 Y23.745 E0.01318 ; infill
G1 X6.234 Y23.745 E0.01123 ; infill
M761
G1 E-2.00000 F0.00000 ; retract
G1 Z3.600 F600.000 ; lift Z
G1 X6.500 Y23.913 F600.000 ; move to first infill point
G1 X6.059 Y27.242 F600.000 ; move to first infill point
G1 X6.046 Y27.745 F600.000 ; move to first infill point
G1 X6.218 Y27.902 F600.000 ; move to first infill point
G1 X6.500 Y31.085 F600.000 ; move to first infill point
G1 X6.755 Y31.252 F600.000 ; move to first infill point
G1 Z1.600 F600.000 ; restore layer Z
M760
G1 E2.00000 F0.00000 ; unretract
G1 X6.238 Y31.252 E0.01093 F600.000 ; infill
G1 X5.732 Y31.626 E0.01328 ; infill
G1 X7.268 Y31.626 E0.03245 ; infill
G1 X7.345 Y31.999 E0.00805 ; infill
G1 X5.655 Y31.999 E0.03568 ; infill
G1 X5.731 Y32.372 E0.00804 ; infill
G1 X7.266 Y32.372 E0.03241 ; infill
G1 X6.766 Y32.745 E0.01318 ; infill
G1 X6.234 Y32.745 E0.01123 ; infill
M761
G1 E-2.00000 F0.00000 ; retract
G1 Z3.600 F600.000 ; lift Z
G1 X6.500 Y31.085 F600.000 ; move to first infill point
G1 X8.274 Y21.966 F600.000 ; move to first infill point
G1 X7.348 Y11.146 F600.000 ; move to first infill point
G1 X8.411 Y5.132 F600.000 ; move to first infill point
G1 X8.137 Y3.546 F600.000 ; move to first infill point
G1 X6.880 Y1.576 F600.000 ; move to first infill point
```

```
G1 X6.046 Y0.745 F600.000 ; move to first infill point
G1 X6.059 Y0.242 F600.000 ; move to first infill point
G1 X6.230 Y0.085 F600.000 ; move to first infill point
G1 X6.500 Y-3.087 F600.000 ; move to first infill point
G1 X6.234 Y-3.255 F600.000 ; move to first infill point
G1 Z1.600 F600.000 ; restore layer Z
M760
G1 E2.00000 F0.00000 ; unretract
G1 X6.766 Y-3.255 E0.01123 F600.000 ; infill
G1 X7.266 Y-3.628 E0.01318 ; infill
G1 X5.730 Y-3.628 E0.03244 ; infill
G1 X5.656 Y-4.001 E0.00803 ; infill
G1 X7.345 Y-4.001 E0.03568 ; infill
G1 X7.268 Y-4.374 E0.00805 ; infill
G1 X5.732 Y-4.374 E0.03245 ; infill
G1 X6.238 Y-4.748 E0.01328 ; infill
G1 X6.756 Y-4.748 E0.01093 ; infill
M761
G1 E-2.00000 F0.00000 ; retract
G1 Z3.600 F600.000 ; lift Z
G1 X6.500 Y-4.915 F600.000 ; move to first infill point
G1 X6.046 Y-8.255 F600.000 ; move to first infill point
G1 X6.059 Y-8.758 F600.000 ; move to first infill point
G1 X6.230 Y-8.915 F600.000 ; move to first infill point
G1 X6.500 Y-12.087 F600.000 ; move to first infill point
G1 X6.234 Y-12.255 F600.000 ; move to first infill point
G1 Z1.600 F600.000 ; restore layer Z
M760
G1 E2.00000 F0.00000 ; unretract
G1 X6.766 Y-12.255 E0.01123 F600.000 ; infill
G1 X7.266 Y-12.628 E0.01318 ; infill
G1 X5.730 Y-12.628 E0.03244 ; infill
G1 X5.656 Y-13.001 E0.00803 ; infill
G1 X7.345 Y-13.001 E0.03568 ; infill
G1 X7.268 Y-13.374 E0.00805 ; infill
G1 X5.732 Y-13.374 E0.03245 ; infill
G1 X6.238 Y-13.748 E0.01328 ; infill
G1 X6.756 Y-13.748 E0.01093 ; infill
M761
G1 E-2.00000 F0.00000 ; retract
G1 Z3.600 F600.000 ; lift Z
G1 X6.500 Y-13.915 F600.000 ; move to first infill point
```

```
G1 X6.046 Y-17.255 F600.000 ; move to first infill point
G1 X6.059 Y-17.758 F600.000 ; move to first infill point
G1 X6.230 Y-17.915 F600.000 ; move to first infill point
G1 X6.500 Y-21.087 F600.000 ; move to first infill point
G1 X6.234 Y-21.255 F600.000 ; move to first infill point
G1 Z1.600 F600.000 ; restore layer Z
M760
G1 E2.00000 F0.00000 ; unretract
G1 X6.766 Y-21.255 E0.01123 F600.000 ; infill
G1 X7.266 Y-21.628 E0.01318 ; infill
G1 X5.730 Y-21.628 E0.03244 ; infill
G1 X5.656 Y-22.001 E0.00803 ; infill
G1 X7.345 Y-22.001 E0.03568 ; infill
G1 X7.268 Y-22.374 E0.00805 ; infill
G1 X5.732 Y-22.374 E0.03245 ; infill
G1 X6.238 Y-22.748 E0.01328 ; infill
G1 X6.755 Y-22.748 E0.01093 ; infill
M761
G1 E-2.00000 F0.00000 ; retract
G1 Z3.600 F600.000 ; lift Z
G1 X5.598 Y-22.152 F600.000 ; move to first infill point
G1 X2.000 Y-22.568 F600.000 ; move to first infill point
G1 X2.000 Y-22.497 F600.000 ; move to first infill point
G1 X-1.590 Y-22.082 F600.000 ; move to first infill point
G1 X-2.245 Y-22.748 F600.000 ; move to first infill point
G1 Z1.600 F600.000 ; restore layer Z
M760
G1 E2.00000 F0.00000 ; unretract
G1 X-2.762 Y-22.748 E0.01093 F600.000 ; infill
G1 X-3.268 Y-22.374 E0.01328 ; infill
G1 X-1.732 Y-22.374 E0.03245 ; infill
G1 X-1.655 Y-22.001 E0.00805 ; infill
G1 X-3.344 Y-22.001 E0.03568 ; infill
G1 X-3.270 Y-21.628 E0.00803 ; infill
G1 X-1.734 Y-21.628 E0.03244 ; infill
G1 X-2.234 Y-21.255 E0.01318 ; infill
G1 X-2.766 Y-21.255 E0.01123 ; infill
M761
G1 E-2.00000 F0.00000 ; retract
G1 Z3.600 F600.000 ; lift Z
G1 X-2.500 Y-21.087 F600.000 ; move to first infill point
G1 X-1.768 Y-17.501 F600.000 ; move to first infill point
```

```
G1 X-2.062 Y-17.240 F600.000 ; move to first infill point
G1 X-2.218 Y-17.098 F600.000 ; move to first infill point
G1 X-2.500 Y-13.915 F600.000 ; move to first infill point
G1 X-2.245 Y-13.748 F600.000 ; move to first infill point
G1 Z1.600 F600.000 ; restore layer Z
M760
G1 E2.00000 F0.00000 ; unretract
G1 X-2.762 Y-13.748 E0.01093 F600.000 ; infill
G1 X-3.268 Y-13.374 E0.01328 ; infill
G1 X-1.732 Y-13.374 E0.03245 ; infill
G1 X-1.655 Y-13.001 E0.00805 ; infill
G1 X-3.344 Y-13.001 E0.03568 ; infill
G1 X-3.270 Y-12.628 E0.00803 ; infill
G1 X-1.734 Y-12.628 E0.03244 ; infill
G1 X-2.234 Y-12.255 E0.01318 ; infill
G1 X-2.766 Y-12.255 E0.01123 ; infill
M761
G1 E-2.00000 F0.00000 ; retract
G1 Z3.600 F600.000 ; lift Z
G1 X-2.500 Y-12.087 F600.000 ; move to first infill point
G1 X-1.768 Y-8.501 F600.000 ; move to first infill point
G1 X-2.062 Y-8.240 F600.000 ; move to first infill point
G1 X-2.218 Y-8.098 F600.000 ; move to first infill point
G1 X-2.500 Y-4.915 F600.000 ; move to first infill point
G1 X-2.245 Y-4.748 F600.000 ; move to first infill point
G1 Z1.600 F600.000 ; restore layer Z
M760
G1 E2.00000 F0.00000 ; unretract
G1 X-2.762 Y-4.748 E0.01093 F600.000 ; infill
G1 X-3.268 Y-4.374 E0.01328 ; infill
G1 X-1.732 Y-4.374 E0.03245 ; infill
G1 X-1.655 Y-4.001 E0.00805 ; infill
G1 X-3.344 Y-4.001 E0.03568 ; infill
G1 X-3.270 Y-3.628 E0.00803 ; infill
G1 X-1.734 Y-3.628 E0.03244 ; infill
G1 X-2.234 Y-3.255 E0.01318 ; infill
G1 X-2.766 Y-3.255 E0.01123 ; infill
M761
G1 E-2.00000 F0.00000 ; retract
G1 Z3.600 F600.000 ; lift Z
G1 X-2.500 Y-4.915 F600.000 ; move to first infill point
G1 X-4.404 Y-12.737 F600.000 ; move to first infill point
```

```
G1 X-4.213 Y-20.763 F600.000 ; move to first infill point
G1 X-4.353 Y-22.677 F600.000 ; move to first infill point
G1 X-3.684 Y-24.359 F600.000 ; move to first infill point
G1 X-2.062 Y-26.240 F600.000 ; move to first infill point
G1 X-1.768 Y-26.501 F600.000 ; move to first infill point
G1 X-2.229 Y-26.915 F600.000 ; move to first infill point
G1 X-2.500 Y-30.087 F600.000 ; move to first infill point
G1 X-2.766 Y-30.255 F600.000 ; move to first infill point
G1 Z1.600 F600.000 ; restore layer Z
M760
G1 E2.00000 F0.00000 ; unretract
G1 X-2.234 Y-30.255 E0.01123 F600.000 ; infill
G1 X-1.734 Y-30.628 E0.01318 ; infill
G1 X-3.270 Y-30.628 E0.03244 ; infill
G1 X-3.344 Y-31.001 E0.00803 ; infill
G1 X-1.655 Y-31.001 E0.03567 ; infill
G1 X-1.732 Y-31.374 E0.00804 ; infill
G1 X-3.268 Y-31.374 E0.03245 ; infill
G1 X-2.762 Y-31.748 E0.01328 ; infill
G1 X-2.245 Y-31.748 E0.01093 ; infill
M761
G1 E-2.00000 F0.00000 ; retract
G1 Z3.600 F600.000 ; lift Z
G1 X-1.587 Y-31.016 F600.000 ; move to first infill point
G1 X1.700 Y-30.610 F600.000 ; move to first infill point
G1 X2.000 Y-30.271 F600.000 ; move to first infill point
G1 X2.083 Y-30.367 F600.000 ; move to first infill point
G1 X5.594 Y-30.884 F600.000 ; move to first infill point
G1 X6.234 Y-30.255 F600.000 ; move to first infill point
G1 Z1.600 F600.000 ; restore layer Z
M760
G1 E2.00000 F0.00000 ; unretract
G1 X6.766 Y-30.255 E0.01123 F600.000 ; infill
G1 X7.266 Y-30.628 E0.01318 ; infill
G1 X5.730 Y-30.628 E0.03244 ; infill
G1 X5.656 Y-31.001 E0.00803 ; infill
G1 X7.345 Y-31.001 E0.03567 ; infill
G1 X7.268 Y-31.374 E0.00804 ; infill
G1 X5.732 Y-31.374 E0.03245 ; infill
G1 X6.238 Y-31.748 E0.01328 ; infill
G1 X6.755 Y-31.748 E0.01093 ; infill
G1 Z2.000 F600.000 ; move to next layer (4)
```

```
M761
G1 E-2.00000 F0.00000 ; retract
G1 Z4.000 F600.000 ; lift Z
G1 X-2.500 Y31.574 F600.000 ; move to first infill point
G1 Z2.000 F600.000 ; restore layer Z
M760
G1 E2.00000 F0.00000 ; unretract
G1 X-2.276 Y31.639 E0.00441 F600.000 ; infill
G1 X-2.178 Y31.723 E0.00243 ; infill
G1 X-2.086 Y31.892 E0.00365 ; infill
G1 X-2.071 Y31.978 E0.00165 ; infill
G1 X-2.106 Y32.164 E0.00358 ; infill
G1 X-2.168 Y32.271 E0.00235 ; infill
G1 X-2.280 Y32.366 E0.00277 ; infill
G1 X-2.388 Y32.413 E0.00223 ; infill
G1 X-2.500 Y32.427 E0.00213 ; infill
G1 X-2.612 Y32.413 E0.00213 ; infill
G1 X-2.721 Y32.365 E0.00225 ; infill
G1 X-2.825 Y32.279 E0.00257 ; infill
G1 X-2.894 Y32.163 E0.00255 ; infill
G1 X-2.925 Y32.067 E0.00190 ; infill
G1 X-2.914 Y31.892 E0.00332 ; infill
G1 X-2.822 Y31.723 E0.00365 ; infill
G1 X-2.720 Y31.635 E0.00254 ; infill
G1 X-2.559 Y31.582 E0.00320 ; infill
M761
G1 E-2.00000 F0.00000 ; retract
G1 Z4.000 F600.000 ; lift Z
G1 X-2.500 Y31.406 F600.000 ; move to first infill point
G1 X-1.907 Y28.268 F600.000 ; move to first infill point
G1 X-1.957 Y26.691 F600.000 ; move to first infill point
G1 X-2.136 Y26.534 F600.000 ; move to first infill point
G1 X-2.500 Y23.595 F600.000 ; move to first infill point
G1 X-2.500 Y22.574 F600.000 ; move to first infill point
G1 Z2.000 F600.000 ; restore layer Z
M760
G1 E2.00000 F0.00000 ; unretract
G1 X-2.276 Y22.639 E0.00441 F600.000 ; infill
G1 X-2.178 Y22.723 E0.00243 ; infill
G1 X-2.086 Y22.892 E0.00365 ; infill
G1 X-2.071 Y22.978 E0.00165 ; infill
G1 X-2.106 Y23.164 E0.00358 ; infill
```

```
G1 X-2.168 Y23.271 E0.00235 ; infill
G1 X-2.280 Y23.366 E0.00277 ; infill
G1 X-2.388 Y23.413 E0.00223 ; infill
G1 X-2.500 Y23.427 E0.00213 ; infill
G1 X-2.612 Y23.413 E0.00213 ; infill
G1 X-2.721 Y23.365 E0.00225 ; infill
G1 X-2.825 Y23.279 E0.00257 ; infill
G1 X-2.894 Y23.163 E0.00255 ; infill
G1 X-2.925 Y23.067 E0.00190 ; infill
G1 X-2.914 Y22.892 E0.00332 ; infill
G1 X-2.822 Y22.723 E0.00365 ; infill
G1 X-2.720 Y22.635 E0.00254 ; infill
G1 X-2.559 Y22.582 E0.00320 ; infill
M761
G1 E-2.00000 F0.00000 ; retract
G1 Z4.000 F600.000 ; lift Z
G1 X-2.500 Y22.406 F600.000 ; move to first infill point
G1 X-1.907 Y19.268 F600.000 ; move to first infill point
G1 X-1.957 Y17.691 F600.000 ; move to first infill point
G1 X-2.136 Y17.534 F600.000 ; move to first infill point
G1 X-2.500 Y14.595 F600.000 ; move to first infill point
G1 X-2.500 Y13.574 F600.000 ; move to first infill point
G1 Z2.000 F600.000 ; restore layer Z
M760
G1 E2.00000 F0.00000 ; unretract
G1 X-2.276 Y13.639 E0.00441 F600.000 ; infill
G1 X-2.178 Y13.723 E0.00243 ; infill
G1 X-2.086 Y13.892 E0.00365 ; infill
G1 X-2.071 Y13.978 E0.00165 ; infill
G1 X-2.106 Y14.164 E0.00358 ; infill
G1 X-2.168 Y14.271 E0.00235 ; infill
G1 X-2.280 Y14.366 E0.00277 ; infill
G1 X-2.388 Y14.413 E0.00223 ; infill
G1 X-2.500 Y14.427 E0.00213 ; infill
G1 X-2.612 Y14.413 E0.00213 ; infill
G1 X-2.721 Y14.365 E0.00225 ; infill
G1 X-2.825 Y14.279 E0.00257 ; infill
G1 X-2.894 Y14.163 E0.00255 ; infill
G1 X-2.925 Y14.067 E0.00190 ; infill
G1 X-2.914 Y13.892 E0.00332 ; infill
G1 X-2.822 Y13.723 E0.00365 ; infill
G1 X-2.720 Y13.635 E0.00254 ; infill
```

```
G1 X-2.559 Y13.582 E0.00320 ; infill
M761
G1 E-2.00000 F0.00000 ; retract
G1 Z4.000 F600.000 ; lift Z
G1 X-2.500 Y13.406 F600.000 ; move to first infill point
G1 X-1.907 Y10.268 F600.000 ; move to first infill point
G1 X-1.957 Y8.691 F600.000 ; move to first infill point
G1 X-2.136 Y8.534 F600.000 ; move to first infill point
G1 X-2.500 Y5.595 F600.000 ; move to first infill point
G1 X-2.500 Y4.574 F600.000 ; move to first infill point
G1 Z2.000 F600.000 ; restore layer Z
M760
G1 E2.00000 F0.00000 ; unretract
G1 X-2.276 Y4.639 E0.00441 F600.000 ; infill
G1 X-2.178 Y4.723 E0.00243 ; infill
G1 X-2.086 Y4.892 E0.00365 ; infill
G1 X-2.071 Y4.978 E0.00165 ; infill
G1 X-2.106 Y5.164 E0.00358 ; infill
G1 X-2.168 Y5.271 E0.00235 ; infill
G1 X-2.280 Y5.366 E0.00277 ; infill
G1 X-2.388 Y5.413 E0.00223 ; infill
G1 X-2.500 Y5.427 E0.00213 ; infill
G1 X-2.612 Y5.413 E0.00213 ; infill
G1 X-2.721 Y5.365 E0.00225 ; infill
G1 X-2.825 Y5.279 E0.00257 ; infill
G1 X-2.894 Y5.163 E0.00255 ; infill
G1 X-2.925 Y5.067 E0.00190 ; infill
G1 X-2.914 Y4.892 E0.00332 ; infill
G1 X-2.822 Y4.723 E0.00365 ; infill
G1 X-2.720 Y4.635 E0.00254 ; infill
G1 X-2.559 Y4.582 E0.00320 ; infill
M761
G1 E-2.00000 F0.00000 ; retract
G1 Z4.000 F600.000 ; lift Z
G1 X-1.903 Y4.968 F600.000 ; move to first infill point
G1 X2.000 Y5.152 F600.000 ; move to first infill point
G1 X2.000 Y5.152 F600.000 ; move to first infill point
G1 X5.903 Y4.968 F600.000 ; move to first infill point
G1 X6.388 Y4.590 F600.000 ; move to first infill point
G1 Z2.000 F600.000 ; restore layer Z
M760
G1 E2.00000 F0.00000 ; unretract
```

```
G1 X6.562 Y4.583 E0.00329 F600.000 ; infill
G1 X6.724 Y4.639 E0.00325 ; infill
G1 X6.822 Y4.723 E0.00243 ; infill
G1 X6.914 Y4.892 E0.00365 ; infill
G1 X6.925 Y5.067 E0.00332 ; infill
G1 X6.832 Y5.271 E0.00424 ; infill
G1 X6.720 Y5.366 E0.00277 ; infill
G1 X6.612 Y5.413 E0.00223 ; infill
G1 X6.500 Y5.427 E0.00213 ; infill
G1 X6.388 Y5.413 E0.00213 ; infill
G1 X6.279 Y5.365 E0.00225 ; infill
G1 X6.175 Y5.279 E0.00257 ; infill
G1 X6.106 Y5.163 E0.00255 ; infill
G1 X6.075 Y5.067 E0.00190 ; infill
G1 X6.086 Y4.892 E0.00332 ; infill
G1 X6.178 Y4.723 E0.00365 ; infill
G1 X6.333 Y4.613 E0.00359 ; infill
M761
G1 E-2.00000 F0.00000 ; retract
G1 Z4.000 F600.000 ; lift Z
G1 X6.500 Y5.595 F600.000 ; move to first infill point
G1 X5.957 Y8.691 F600.000 ; move to first infill point
G1 X5.907 Y10.268 F600.000 ; move to first infill point
G1 X6.088 Y10.426 F600.000 ; move to first infill point
G1 X6.500 Y13.406 F600.000 ; move to first infill point
G1 X6.388 Y13.590 F600.000 ; move to first infill point
G1 Z2.000 F600.000 ; restore layer Z
M760
G1 E2.00000 F0.00000 ; unretract
G1 X6.562 Y13.583 E0.00329 F600.000 ; infill
G1 X6.724 Y13.639 E0.00325 ; infill
G1 X6.822 Y13.723 E0.00243 ; infill
G1 X6.914 Y13.892 E0.00365 ; infill
G1 X6.925 Y14.067 E0.00332 ; infill
G1 X6.832 Y14.271 E0.00424 ; infill
G1 X6.720 Y14.366 E0.00277 ; infill
G1 X6.612 Y14.413 E0.00223 ; infill
G1 X6.500 Y14.427 E0.00213 ; infill
G1 X6.388 Y14.413 E0.00213 ; infill
G1 X6.279 Y14.365 E0.00225 ; infill
G1 X6.175 Y14.279 E0.00257 ; infill
G1 X6.106 Y14.163 E0.00255 ; infill
```

```
G1 X6.075 Y14.067 E0.00190 ; infill
G1 X6.086 Y13.892 E0.00332 ; infill
G1 X6.178 Y13.723 E0.00365 ; infill
G1 X6.333 Y13.613 E0.00359 ; infill
M761
G1 E-2.00000 F0.00000 ; retract
G1 Z4.000 F600.000 ; lift Z
G1 X6.500 Y14.595 F600.000 ; move to first infill point
G1 X5.957 Y17.691 F600.000 ; move to first infill point
G1 X5.907 Y19.268 F600.000 ; move to first infill point
G1 X6.088 Y19.426 F600.000 ; move to first infill point
G1 X6.500 Y22.406 F600.000 ; move to first infill point
G1 X6.388 Y22.590 F600.000 ; move to first infill point
G1 Z2.000 F600.000 ; restore layer Z
M760
G1 E2.00000 F0.00000 ; unretract
G1 X6.562 Y22.583 E0.00329 F600.000 ; infill
G1 X6.724 Y22.639 E0.00325 ; infill
G1 X6.822 Y22.723 E0.00243 ; infill
G1 X6.914 Y22.892 E0.00365 ; infill
G1 X6.925 Y23.067 E0.00332 ; infill
G1 X6.832 Y23.271 E0.00424 ; infill
G1 X6.720 Y23.366 E0.00277 ; infill
G1 X6.612 Y23.413 E0.00223 ; infill
G1 X6.500 Y23.427 E0.00213 ; infill
G1 X6.388 Y23.413 E0.00213 ; infill
G1 X6.279 Y23.365 E0.00225 ; infill
G1 X6.175 Y23.279 E0.00257 ; infill
G1 X6.106 Y23.163 E0.00255 ; infill
G1 X6.075 Y23.067 E0.00190 ; infill
G1 X6.086 Y22.892 E0.00332 ; infill
G1 X6.178 Y22.723 E0.00365 ; infill
G1 X6.333 Y22.613 E0.00359 ; infill
M761
G1 E-2.00000 F0.00000 ; retract
G1 Z4.000 F600.000 ; lift Z
G1 X6.500 Y23.595 F600.000 ; move to first infill point
G1 X5.957 Y26.691 F600.000 ; move to first infill point
G1 X5.907 Y28.268 F600.000 ; move to first infill point
G1 X6.088 Y28.426 F600.000 ; move to first infill point
G1 X6.500 Y31.406 F600.000 ; move to first infill point
G1 X6.388 Y31.590 F600.000 ; move to first infill point
```

```
G1 Z2.000 F600.000 ; restore layer Z
M760
G1 E2.00000 F0.00000 ; unretract
G1 X6.562 Y31.583 E0.00329 F600.000 ; infill
G1 X6.724 Y31.639 E0.00325 ; infill
G1 X6.822 Y31.723 E0.00243 ; infill
G1 X6.914 Y31.892 E0.00365 ; infill
G1 X6.925 Y32.067 E0.00332 ; infill
G1 X6.832 Y32.271 E0.00424 ; infill
G1 X6.720 Y32.366 E0.00277 ; infill
G1 X6.612 Y32.413 E0.00223 ; infill
G1 X6.500 Y32.427 E0.00213 ; infill
G1 X6.388 Y32.413 E0.00213 ; infill
G1 X6.279 Y32.365 E0.00225 ; infill
G1 X6.175 Y32.279 E0.00257 ; infill
G1 X6.106 Y32.163 E0.00255 ; infill
G1 X6.075 Y32.067 E0.00190 ; infill
G1 X6.086 Y31.892 E0.00332 ; infill
G1 X6.178 Y31.723 E0.00365 ; infill
G1 X6.333 Y31.613 E0.00359 ; infill
M761
G1 E-2.00000 F0.00000 ; retract
G1 Z4.000 F600.000 ; lift Z
G1 X6.500 Y31.406 F600.000 ; move to first infill point
G1 X2.000 Y15.016 F600.000 ; move to first infill point
G1 X2.000 Y6.015 F600.000 ; move to first infill point
G1 X2.000 Y0.503 F600.000 ; move to first infill point
G1 X4.393 Y0.501 F600.000 ; move to first infill point
G1 X4.858 Y0.501 F600.000 ; move to first infill point
G1 X5.957 Y-0.308 F600.000 ; move to first infill point
G1 X6.135 Y-0.466 F600.000 ; move to first infill point
G1 X6.500 Y-3.405 F600.000 ; move to first infill point
G1 X6.388 Y-4.410 F600.000 ; move to first infill point
G1 Z2.000 F600.000 ; restore layer Z
M760
G1 E2.00000 F0.00000 ; unretract
G1 X6.562 Y-4.417 E0.00329 F600.000 ; infill
G1 X6.724 Y-4.361 E0.00325 ; infill
G1 X6.822 Y-4.277 E0.00243 ; infill
G1 X6.914 Y-4.108 E0.00365 ; infill
G1 X6.925 Y-3.933 E0.00332 ; infill
G1 X6.832 Y-3.729 E0.00424 ; infill
```

```
G1 X6.720 Y-3.634 E0.00277 ; infill
G1 X6.612 Y-3.587 E0.00223 ; infill
G1 X6.500 Y-3.573 E0.00213 ; infill
G1 X6.388 Y-3.587 E0.00213 ; infill
G1 X6.279 Y-3.635 E0.00225 ; infill
G1 X6.175 Y-3.721 E0.00257 ; infill
G1 X6.106 Y-3.837 E0.00255 ; infill
G1 X6.075 Y-3.933 E0.00190 ; infill
G1 X6.086 Y-4.108 E0.00332 ; infill
G1 X6.178 Y-4.277 E0.00365 ; infill
G1 X6.333 Y-4.387 E0.00359 ; infill
M761
G1 E-2.00000 F0.00000 ; retract
G1 Z4.000 F600.000 ; lift Z
G1 X6.500 Y-4.594 F600.000 ; move to first infill point
G1 X5.907 Y-7.732 F600.000 ; move to first infill point
G1 X5.957 Y-9.308 F600.000 ; move to first infill point
G1 X6.135 Y-9.466 F600.000 ; move to first infill point
G1 X6.500 Y-12.405 F600.000 ; move to first infill point
G1 X6.280 Y-13.365 F600.000 ; move to first infill point
G1 Z2.000 F600.000 ; restore layer Z
M760
G1 E2.00000 F0.00000 ; unretract
G1 X6.388 Y-13.410 E0.00222 F600.000 ; infill
G1 X6.562 Y-13.417 E0.00329 ; infill
G1 X6.724 Y-13.361 E0.00325 ; infill
G1 X6.822 Y-13.277 E0.00243 ; infill
G1 X6.914 Y-13.108 E0.00365 ; infill
G1 X6.925 Y-12.933 E0.00332 ; infill
G1 X6.832 Y-12.729 E0.00424 ; infill
G1 X6.720 Y-12.634 E0.00277 ; infill
G1 X6.612 Y-12.587 E0.00223 ; infill
G1 X6.500 Y-12.573 E0.00213 ; infill
G1 X6.388 Y-12.587 E0.00213 ; infill
G1 X6.279 Y-12.635 E0.00225 ; infill
G1 X6.175 Y-12.721 E0.00257 ; infill
G1 X6.106 Y-12.837 E0.00255 ; infill
G1 X6.071 Y-13.022 E0.00356 ; infill
G1 X6.086 Y-13.108 E0.00165 ; infill
G1 X6.178 Y-13.277 E0.00365 ; infill
G1 X6.234 Y-13.326 E0.00141 ; infill
M761
```

```
G1 E-2.00000 F0.00000 ; retract
G1 Z4.000 F600.000 ; lift Z
G1 X6.500 Y-13.594 F600.000 ; move to first infill point
G1 X5.907 Y-16.732 F600.000 ; move to first infill point
G1 X5.957 Y-18.308 F600.000 ; move to first infill point
G1 X6.135 Y-18.466 F600.000 ; move to first infill point
G1 X6.500 Y-21.405 F600.000 ; move to first infill point
G1 X6.178 Y-22.277 F600.000 ; move to first infill point
G1 Z2.000 F600.000 ; restore layer Z
M760
G1 E2.00000 F0.00000 ; unretract
G1 X6.280 Y-22.365 E0.00254 F600.000 ; infill
G1 X6.388 Y-22.410 E0.00222 ; infill
G1 X6.562 Y-22.417 E0.00329 ; infill
G1 X6.724 Y-22.361 E0.00325 ; infill
G1 X6.822 Y-22.277 E0.00243 ; infill
G1 X6.914 Y-22.108 E0.00365 ; infill
G1 X6.929 Y-22.022 E0.00165 ; infill
G1 X6.894 Y-21.836 E0.00358 ; infill
G1 X6.832 Y-21.729 E0.00235 ; infill
G1 X6.720 Y-21.634 E0.00277 ; infill
G1 X6.612 Y-21.587 E0.00223 ; infill
G1 X6.500 Y-21.573 E0.00213 ; infill
G1 X6.388 Y-21.587 E0.00213 ; infill
G1 X6.279 Y-21.635 E0.00225 ; infill
G1 X6.175 Y-21.721 E0.00257 ; infill
G1 X6.106 Y-21.837 E0.00255 ; infill
G1 X6.075 Y-21.933 E0.00190 ; infill
G1 X6.086 Y-22.108 E0.00332 ; infill
G1 X6.149 Y-22.225 E0.00251 ; infill
M761
G1 E-2.00000 F0.00000 ; retract
G1 Z4.000 F600.000 ; lift Z
G1 X5.903 Y-22.032 F600.000 ; move to first infill point
G1 X2.000 Y-21.848 F600.000 ; move to first infill point
G1 X2.000 Y-21.848 F600.000 ; move to first infill point
G1 X-1.903 Y-22.032 F600.000 ; move to first infill point
G1 X-2.500 Y-22.426 F600.000 ; move to first infill point
G1 Z2.000 F600.000 ; restore layer Z
M760
G1 E2.00000 F0.00000 ; unretract
G1 X-2.276 Y-22.361 E0.00441 F600.000 ; infill
```

```
G1 X-2.178 Y-22.277 E0.00243 ; infill
G1 X-2.086 Y-22.108 E0.00365 ; infill
G1 X-2.071 Y-22.022 E0.00165 ; infill
G1 X-2.106 Y-21.836 E0.00358 ; infill
G1 X-2.168 Y-21.729 E0.00235 ; infill
G1 X-2.280 Y-21.634 E0.00277 ; infill
G1 X-2.388 Y-21.587 E0.00223 ; infill
G1 X-2.500 Y-21.573 E0.00213 ; infill
G1 X-2.612 Y-21.587 E0.00213 ; infill
G1 X-2.721 Y-21.635 E0.00225 ; infill
G1 X-2.825 Y-21.721 E0.00257 ; infill
G1 X-2.894 Y-21.837 E0.00255 ; infill
G1 X-2.925 Y-21.933 E0.00190 ; infill
G1 X-2.914 Y-22.108 E0.00332 ; infill
G1 X-2.822 Y-22.277 E0.00365 ; infill
G1 X-2.720 Y-22.365 E0.00254 ; infill
G1 X-2.559 Y-22.418 E0.00320 ; infill
M761
G1 E-2.00000 F0.00000 ; retract
G1 Z4.000 F600.000 ; lift Z
G1 X-2.500 Y-21.405 F600.000 ; move to first infill point
G1 X-0.921 Y-17.499 F600.000 ; move to first infill point
G1 X-1.971 Y-16.676 F600.000 ; move to first infill point
G1 X-2.089 Y-16.574 F600.000 ; move to first infill point
G1 X-2.500 Y-13.594 F600.000 ; move to first infill point
G1 X-2.500 Y-13.426 F600.000 ; move to first infill point
G1 Z2.000 F600.000 ; restore layer Z
M760
G1 E2.00000 F0.00000 ; unretract
G1 X-2.276 Y-13.361 E0.00441 F600.000 ; infill
G1 X-2.178 Y-13.277 E0.00243 ; infill
G1 X-2.086 Y-13.108 E0.00365 ; infill
G1 X-2.071 Y-13.022 E0.00165 ; infill
G1 X-2.106 Y-12.836 E0.00358 ; infill
G1 X-2.168 Y-12.729 E0.00235 ; infill
G1 X-2.280 Y-12.634 E0.00277 ; infill
G1 X-2.388 Y-12.587 E0.00223 ; infill
G1 X-2.500 Y-12.573 E0.00213 ; infill
G1 X-2.612 Y-12.587 E0.00213 ; infill
G1 X-2.721 Y-12.635 E0.00225 ; infill
G1 X-2.825 Y-12.721 E0.00257 ; infill
G1 X-2.894 Y-12.837 E0.00255 ; infill
```

```
G1 X-2.925 Y-12.933 E0.00190 ; infill
G1 X-2.914 Y-13.108 E0.00332 ; infill
G1 X-2.822 Y-13.277 E0.00365 ; infill
G1 X-2.720 Y-13.365 E0.00254 ; infill
G1 X-2.559 Y-13.418 E0.00320 ; infill
M761
G1 E-2.00000 F0.00000 ; retract
G1 Z4.000 F600.000 ; lift Z
G1 X-2.500 Y-12.405 F600.000 ; move to first infill point
G1 X-0.921 Y-8.499 F600.000 ; move to first infill point
G1 X-1.971 Y-7.676 F600.000 ; move to first infill point
G1 X-2.089 Y-7.574 F600.000 ; move to first infill point
G1 X-2.500 Y-4.594 F600.000 ; move to first infill point
G1 X-2.500 Y-4.426 F600.000 ; move to first infill point
G1 Z2.000 F600.000 ; restore layer Z
M760
G1 E2.00000 F0.00000 ; unretract
G1 X-2.276 Y-4.361 E0.00441 F600.000 ; infill
G1 X-2.178 Y-4.277 E0.00243 ; infill
G1 X-2.086 Y-4.108 E0.00365 ; infill
G1 X-2.071 Y-4.022 E0.00165 ; infill
G1 X-2.106 Y-3.836 E0.00358 ; infill
G1 X-2.168 Y-3.729 E0.00235 ; infill
G1 X-2.280 Y-3.634 E0.00277 ; infill
G1 X-2.388 Y-3.587 E0.00223 ; infill
G1 X-2.500 Y-3.573 E0.00213 ; infill
G1 X-2.612 Y-3.587 E0.00213 ; infill
G1 X-2.721 Y-3.635 E0.00225 ; infill
G1 X-2.825 Y-3.721 E0.00257 ; infill
G1 X-2.894 Y-3.837 E0.00255 ; infill
G1 X-2.925 Y-3.933 E0.00190 ; infill
G1 X-2.914 Y-4.108 E0.00332 ; infill
G1 X-2.822 Y-4.277 E0.00365 ; infill
G1 X-2.720 Y-4.365 E0.00254 ; infill
G1 X-2.559 Y-4.418 E0.00320 ; infill
M761
G1 E-2.00000 F0.00000 ; retract
G1 Z4.000 F600.000 ; lift Z
G1 X-2.500 Y-4.594 F600.000 ; move to first infill point
G1 X-4.067 Y-12.571 F600.000 ; move to first infill point
G1 X-3.788 Y-20.601 F600.000 ; move to first infill point
G1 X-3.949 Y-22.963 F600.000 ; move to first infill point
```

```
G1 X-2.812 Y-24.857 F600.000 ; move to first infill point
G1 X-1.907 Y-25.732 F600.000 ; move to first infill point
G1 X-1.957 Y-27.308 F600.000 ; move to first infill point
G1 X-2.135 Y-27.466 F600.000 ; move to first infill point
G1 X-2.500 Y-30.405 F600.000 ; move to first infill point
G1 X-2.822 Y-31.277 F600.000 ; move to first infill point
G1 Z2.000 F600.000 ; restore layer Z
M760
G1 E2.00000 F0.00000 ; unretract
G1 X-2.720 Y-31.365 E0.00254 F600.000 ; infill
G1 X-2.612 Y-31.410 E0.00222 ; infill
G1 X-2.438 Y-31.417 E0.00329 ; infill
G1 X-2.276 Y-31.361 E0.00325 ; infill
G1 X-2.178 Y-31.277 E0.00243 ; infill
G1 X-2.086 Y-31.108 E0.00365 ; infill
G1 X-2.071 Y-31.022 E0.00165 ; infill
G1 X-2.106 Y-30.836 E0.00358 ; infill
G1 X-2.168 Y-30.729 E0.00235 ; infill
G1 X-2.280 Y-30.634 E0.00277 ; infill
G1 X-2.388 Y-30.587 E0.00223 ; infill
G1 X-2.500 Y-30.573 E0.00213 ; infill
G1 X-2.612 Y-30.587 E0.00213 ; infill
G1 X-2.721 Y-30.635 E0.00225 ; infill
G1 X-2.825 Y-30.721 E0.00257 ; infill
G1 X-2.894 Y-30.837 E0.00255 ; infill
G1 X-2.925 Y-30.933 E0.00190 ; infill
G1 X-2.914 Y-31.108 E0.00332 ; infill
G1 X-2.851 Y-31.225 E0.00251 ; infill
M761
G1 E-2.00000 F0.00000 ; retract
G1 Z4.000 F600.000 ; lift Z
G1 X-1.903 Y-31.032 F600.000 ; move to first infill point
G1 X1.947 Y-29.491 F600.000 ; move to first infill point
G1 X2.500 Y-30.090 F600.000 ; move to first infill point
G1 X3.087 Y-30.769 F600.000 ; move to first infill point
G1 X3.217 Y-30.905 F600.000 ; move to first infill point
G1 X5.903 Y-31.032 F600.000 ; move to first infill point
G1 X6.071 Y-31.022 F600.000 ; move to first infill point
G1 Z2.000 F600.000 ; restore layer Z
M760
G1 E2.00000 F0.00000 ; unretract
G1 X6.086 Y-31.108 E0.00165 F600.000 ; infill
```

```
G1 X6.178 Y-31.277 E0.00365 ; infill
G1 X6.280 Y-31.365 E0.00254 ; infill
G1 X6.388 Y-31.410 E0.00222 ; infill
G1 X6.500 Y-31.426 E0.00213 ; infill
G1 X6.724 Y-31.361 E0.00441 ; infill
G1 X6.822 Y-31.277 E0.00243 ; infill
G1 X6.914 Y-31.108 E0.00365 ; infill
G1 X6.929 Y-31.022 E0.00165 ; infill
G1 X6.894 Y-30.836 E0.00358 ; infill
G1 X6.832 Y-30.729 E0.00235 ; infill
G1 X6.720 Y-30.634 E0.00277 ; infill
G1 X6.612 Y-30.587 E0.00223 ; infill
G1 X6.500 Y-30.573 E0.00213 ; infill
G1 X6.388 Y-30.587 E0.00213 ; infill
G1 X6.279 Y-30.635 E0.00225 ; infill
G1 X6.175 Y-30.721 E0.00257 ; infill
G1 X6.106 Y-30.837 E0.00255 ; infill
G1 X6.074 Y-30.962 E0.00244 ; infill
M761
G1 E-2.00000 F0.00000 ; retract
G1 Z4.000 F600.000 ; lift Z
G0 Z10
M84 ;disable motors
;Post processed by INKREDIBLE post processor
```

Supplementary Code File 2 – Example gcode for printing 2 columns worth of organoids in a 96-well plate using the Allevi Allevi2 bioprinter.

```
; generated by Slic3r 1.3.1-dev on 2018-06-22 at 15:39:54

; external perimeters extrusion width = 0.37mm (1.02mm^3/s)
; perimeters extrusion width = 0.36mm (0.97mm^3/s)
; infill extrusion width = 0.36mm (0.97mm^3/s)
; solid infill extrusion width = 0.36mm (0.97mm^3/s)
; top infill extrusion width = 0.36mm (0.97mm^3/s)

M104 S200 ; set temperature
G28 ; home all axes
; Filament gcode

M109 S200 ; set temperature and wait for it to be reached
G21 ; set units to millimeters
```

```
G90 ; use absolute coordinates
M82 ; use absolute distances for extrusion
G92 E0
G1 E-10.00000 F1800.00000
G92 E0
G1 Z8.600 F1200.000
G1 X-0.654 Y-0.743 F1200.000
G1 Z0.500 F1200.000
G1 E20.00000 F1800.00000
G1 F600
G1 X-0.506 Y-0.851 E20.00085
G1 X-0.341 Y-0.929 E20.00170
G1 X-0.164 Y-0.976 E20.00255
G1 X0.049 Y-0.989 E20.00354
G1 X0.236 Y-0.962 E20.00441
G1 X0.409 Y-0.902 E20.00526
G1 X0.568 Y-0.811 E20.00611
G1 X0.733 Y-0.666 E20.00713
G1 X0.843 Y-0.520 E20.00798
G1 X0.937 Y-0.320 E20.00900
G1 X0.981 Y-0.137 E20.00987
G1 X0.989 Y0.054 E20.01076
G1 X0.958 Y0.249 E20.01168
G1 X0.895 Y0.422 E20.01253
G1 X0.780 Y0.610 E20.01355
G1 X0.654 Y0.743 E20.01440
G1 X0.506 Y0.851 E20.01525
G1 X0.341 Y0.929 E20.01610
G1 X0.164 Y0.976 E20.01695
G1 X-0.055 Y0.988 E20.01797
G1 X-0.236 Y0.961 E20.01882
G1 X-0.409 Y0.902 E20.01966
G1 X-0.597 Y0.789 E20.02068
G1 X-0.733 Y0.666 E20.02153
G1 X-0.862 Y0.488 E20.02255
G1 X-0.937 Y0.320 E20.02341
G1 X-0.981 Y0.137 E20.02428
G1 X-0.989 Y-0.054 E20.02516
G1 X-0.958 Y-0.249 E20.02608
G1 X-0.895 Y-0.422 E20.02694
G1 X-0.802 Y-0.580 E20.02779
G1 X-0.688 Y-0.705 E20.02857
```

```
G1 E10.02857 F1800.00000
G92 E0
G1 Z10.500 F1200.000
; Filament-specific end gcode
;END gcode for filament


M104 S0 ; set temperature
; filament used = 10.0mm (0.1cm3)
; total filament cost = 0.0

; avoid_crossing_perimeters = 0
; bed_shape = -90x-90,90x-90,90x90,-90x90
; bed_temperature = 0
; before_layer_gcode =
; between_objects_gcode =
; bridge_acceleration = 0
; bridge_fan_speed = 100
; brim_connections_width = 0
; brim_width = 0
; complete_objects = 1
; cooling = 0
; default_acceleration = 0
; disable_fan_first_layers = 1
; duplicate_distance = 6
; end_filament_gcode = "; Filament-specific end gcode \n;END gcode for filament\n"
; end_gcode =
; extruder_clearance_height = 10
; extruder_clearance_radius = 3
; extruder_offset = 0x0,0x0
; extrusion_axis = E
; extrusion_multiplier = 1
; fan_always_on = 0
; fan_below_layer_time = 60
; filament_colour = #FFFFFF
; filament_cost = 0
; filament_density = 0
; filament_diameter = 3
; filament_max_volumetric_speed = 0
; filament_notes = ""
; first_layer_acceleration = 0
; first_layer_bed_temperature = 0
```

```
; first_layer_extrusion_width = 0
; first_layer_speed = 10
; first_layer_temperature = 200
; gcode_arcs = 0
; gcode_comments = 0
; gcode_flavor = reprap
; has_heatbed = 0
; infill_acceleration = 0
; infill_first = 0
; interior_brim_width = 0
; layer_gcode =
; max_fan_speed = 100
; max_layer_height = 0.3,0.3
; max_print_speed = 80
; max_volumetric_speed = 0
; min_fan_speed = 35
; min_layer_height = 0.15,0.15
; min_print_speed = 10
; min_skirt_length = 0
; notes =
; nozzle_diameter = 0.34,0.1
; only_retract_when_crossing_perimeters = 0
; ooze_prevention = 0
; output_filename_format = [input_filename_base].gcode
; perimeter_acceleration = 0
; post_process =
; pressure_advance = 0
; printer_notes =
; resolution = 0
; retract_before_travel = 0,0
; retract_layer_change = 1,1
; retract_length = 10,10
; retract_length_toolchange = 10,10
; retract_lift = 10,10
; retract_lift_above = -100,-100
; retract_lift_below = 100,100
; retract_restart_extra = 10,10
; retract_restart_extra_toolchange = 10,10
; retract_speed = 30,30
; skirt_distance = 6
; skirt_height = 1
; skirts = 0
```

; slowdown_below_layer_time = 30
; spiral_vase = 0
; standby_temperature_delta = -5
; start_filament_gcode = "; Filament gcode\n"
; start_gcode = G28 ; home all axes
; temperature = 200
; threads = 4
; toolchange_gcode =
; travel_speed = 20
; use_firmware_retraction = 0
; use_relative_e_distances = 0
; use_set_and_wait_bed = 0
; use_set_and_wait_extruder = 0
; use_volumetric_e = 0
; vibration_limit = 0
; wipe = 0,0
; z_offset = -1.4
; z_steps_per_mm = 0
; adaptive_slicing = 0
; adaptive_slicing_quality = 75%
; dont_support_bridges = 1
; extrusion_width = 0
; first_layer_height = 1.9
; infill_only_where_needed = 0
; interface_shells = 0
; layer_height = 1.9
; match_horizontal_surfaces = 0
; raft_layers = 0
; regions_overlap = 0
; seam_position = random
; sequential_print_priority = 0
; support_material = 0
; support_material_angle = 0
; support_material_buildplate_only = 0
; support_material_contact_distance = 0.2
; support_material_enforce_layers = 0
; support_material_extruder = 1
; support_material_extrusion_width = 0
; support_material_interface_extruder = 1
; support_material_interface_extrusion_width = 0
; support_material_interface_layers = 3
; support_material_interface_spacing = 0

; support_material_interface_speed = 100%

; support_material_max_layers = 0

; support_material_pattern = pillars

; support_material_pillar_size = 2.5

; support_material_pillar_spacing = 10

; support_material_spacing = 2.5

; support_material_speed = 60

; support_material_threshold = 60%

; xy_size_compensation = 0

; bottom_infill_pattern = rectilinear

; bottom_solid_layers = 0

; bridge_flow_ratio = 1

; bridge_speed = 10

; external_perimeter_extrusion_width = 0

; external_perimeter_speed = 10

; external_perimeters_first = 0

; extra_perimeters = 0

; fill_angle = 90

; fill_density = 1%

; fill_gaps = 0

; fill_pattern = concentric

; gap_fill_speed = 1

; infill_every_layers = 100

; infill_extruder = 1

; infill_extrusion_width = 0

; infill_overlap = 0

; infill_speed = 10

; overhangs = 1

; perimeter_extruder = 1

; perimeter_extrusion_width = 0

; perimeter_speed = 10

; perimeters = 0

; small_perimeter_speed = 10

; solid_infill_below_area = 0

; solid_infill_every_layers = 0

; solid_infill_extruder = 1

; solid_infill_extrusion_width = 0

; solid_infill_speed = 10

; thin_walls = 1

; top_infill_extrusion_width = 0

; top_infill_pattern = rectilinear

; top_solid_infill_speed = 10

; top_solid_layers = 0
